# Synthesis of 4-Hydroxyphenylamino-Naphthoquinones as Paracetamol-Inspired Analogs: Chemical, In Silico, and Phenotypic Pharmacological Evaluation

**DOI:** 10.3390/pharmaceutics18040482

**Published:** 2026-04-14

**Authors:** Iván M. Quispe-Díaz, Oswaldo Rebaza-Rioja, Sussan Lopez-Mercado, Cinthya Enriquez-Lara, Daniel Asunción-Alvarez, Roberto O. Ybañez-Julca, Elena Mantilla-Rodríguez, Wilfredo O. Gutiérrez-Alvarado, Ricardo Pino-Rios, Jaime A. Valderrama, Julio Benites

**Affiliations:** 1 Facultad de Farmacia y Bioquímica, Universidad Nacional de Trujillo, Trujillo 13011, Peru; orebaza@unitru.edu.pe (O.R.-R.); hasuncion@unitru.edu.pe (D.A.-A.); rybanez@unitru.edu.pe (R.O.Y.-J.); amantilla@unitru.edu.pe (E.M.-R.); 2 Laboratorio de Química Medicinal, Química y Farmacia, Facultad de Ciencias de la Salud, Universidad Arturo Prat, Casilla 121, Iquique 1100000, Chile; sussan.j.lopez.m@gmail.com (S.L.-M.); jaimeadolfov@gmail.com (J.A.V.); 3 Programa de Doctorado en Química Medicinal, Facultad de Ciencias de la Salud, Universidad Arturo Prat, Casilla 121, Iquique 1100000, Chile; cenriquez@estudiantesunap.cl; 4 Facultad de Farmacia y Bioquímica, Universidad Nacional de la Amazonía Peruana, Iquitos 16001, Peru; wilfredo.gutierrez@unapiquitos.edu.pe; 5 Departamento de Ciencias Químicas, Facultad de Ciencias Exactas, Universidad Andrés Bello, Santiago 8370146, Chile; ricardo.pino.r@unab.cl

**Keywords:** 4-hydroxyphenylamino-naphthoquinones, solvent-free synthesis, phenotypic pharmacology, inflammatory pain, antinociceptive activity, paracetamol-inspired analogs

## Abstract

**Background/Objectives**: Paracetamol is a widely analgesic and antipyretic drug; however, its limited anti-inflammatory efficacy and safety concerns motivate the search for novel non-opioid alternatives. In this study, a series of 4-hydroxyphenylamino-naphthoquinones were designed as paracetamol-inspired analogs and synthesized via a solvent-free, silica-assisted Michael addition, providing a sustainable and efficient synthetic route. **Methods**: The compounds were evaluated using an integrated strategy combining in silico prediction, density functional theory calculations, molecular docking, ADMET profiling, and in vivo phenotypic pharmacological assays. **Results:** In vivo evaluation revealed pronounced peripheral antinociceptive activity in the acetic acid-induced writhing model and robust anti-inflammatory effects in carrageenan-induced paw edema, comparable to those of naproxen. These findings suggest a predominantly peripheral mechanism consistent with anti-inflammatory and antinociceptive profiles linked to cyclooxygenase inhibition. A normalization-based multi-criteria analysis integrating peripheral, anti-inflammatory, central, and antipyretic endpoints enabled transparent phenotypic prioritization within the series. Under this framework, compound **7** emerged as the most balanced peripheral–anti-inflammatory candidate, whereas compound **8**, evaluated experimentally as a regioisomeric mixture, showed comparatively stronger central antinociceptive activity in the hot plate test. Antipyretic activity in an LPS-induced fever model was limited and not sustained. **Conclusions**: Overall, these findings indicated that the 4-hydroxyphenylamino-naphthoquinone scaffold emerges as a promising non-opioid platform for peripheral inflammatory pain, supporting further investigation of its pharmacological and mechanistic properties.

## 1. Introduction

In recent years, the consumption of non-steroidal anti-inflammatory drugs (NSAIDs) and paracetamol (PCM) has markedly increased, particularly since the COVID-19 pandemic, owing to their widespread use in the symptomatic treatment of various conditions [1,2]. Unlike NSAIDs, PCM lacks significant anti-inflammatory activity but exhibits well-established analgesic and antipyretic effects. Owing to its efficacy and relative safety at therapeutic doses, PCM has become the most widely used over-the-counter non-narcotic analgesic and remains the first-line therapy for fever and acute pain [3]. 

Despite this favorable safety profile, increasing evidence indicates that inappropriate dosing regimens may shift PCM-related adverse effects from mild to clinically severe [3,4]. Daily doses exceeding 4 g are associated with an increased risk of toxicity [5], whereas chronic intake has been linked to hepatotoxicity, particularly in vulnerable populations such as individuals with chronic alcohol consumption or concomitant use of enzyme-inducing drugs [6]. PCM accounts for up to 46% of cases of acute liver failure in Western countries [7,8], mainly due to its hepatic bioactivation to the reactive metabolite *N*-acetyl-*p*-benzoquinone imine (NAPQI), which induces glutathione depletion, mitochondrial dysfunction, and oxidative stress-mediated hepatocellular injury [9,10,11].

Originally, the analgesic action of PCM was attributed to cyclooxygenase inhibition [12]. However, accumulating evidence has revealed a more complex pharmacological profile involving the modulation of transient receptor potential (TRP) channels, voltage-gated ion channels, and nitric oxide-related pathways [13]. At the central level, PCM is metabolized to *p*-aminophenol, which is further converted to *N*-arachidonoylphenolamine (AM404), a metabolite that interacts with TRPV1 and cannabinoid CB1 receptors [14,15]. Importantly, the compounds described in this study were not designed to mimic the metabolic conversion of paracetamol to AM404, and no mechanistic equivalence with this pathway is assumed. While these findings have expanded the understanding of PCM pharmacology, AM404 is a transient metabolite formed in vivo and is not directly administered, limiting its relevance as a comparator for the rational design of new synthetic compounds.

Based on these considerations, recent efforts have focused on developing paracetamol-inspired analogs with improved pharmacological profiles. Structural modifications of the 4-hydroxyaniline scaffold through bioisosteric replacement and molecular derivatization have yielded compounds with analgesic, antipyretic, and anti-inflammatory activities, often accompanied by reduced hepatotoxicity [16,17,18]. In this context, the term “paracetamol-inspired” in the present study refers to the incorporation of the 4-hydroxyaniline pharmacophore and the pursuit of non-opioid analgesic activity without assuming mechanistic equivalence with paracetamol or its metabolites.

Among potential scaffolds, naphthoquinones are attractive candidates owing to their structural versatility and redox-active nature; however, their systematic exploration as scaffolds for inflammation-driven analgesia incorporating the 4-hydroxyaniline motif remains limited, despite their extensive investigation of diverse biological activities [19,20]. Amino-substituted naphthoquinones have been reported; however, their pharmacological evaluation has predominantly focused on cytotoxic or redox-related activities [21,22], whereas their systematic investigation in inflammation-driven analgesic models remains limited.

In parallel, the growing emphasis on sustainable chemistry has promoted solvent-free and mechanochemical approaches, such as grindstone chemistry, as efficient and environmentally benign synthetic strategies [23,24,25,26]. These methodologies offer advantages including reduced environmental impact, improved reaction efficiency, and enhanced reproducibility [19], supporting their application as sustainable approaches for the synthesis of bioactive compounds. 

Accordingly, the present study was designed to investigate novel paracetamol-inspired compounds with a focus on their phenotypic pharmacological profile in inflammation-related pain models. The working hypothesis is that these compounds exert their pharmacological effects predominantly through peripheral mechanisms associated with inflammatory and nociceptive processes. A series of 4-hydroxyphenylamino-naphthoquinones were synthesized under solvent-free conditions and evaluated using an integrated approach combining in silico analysis and in vivo pharmacological assays.

## 2. Materials and Methods

### 2.1. Chemistry

#### 2.1.1. General

All solvents and reagents were purchased from commercial suppliers, including Aldrich (St. Louis, MO, USA) and Merck (Darmstadt, Germany) and were used as supplied; detailed information (CAS numbers and purity) is provided in Table S1 in the Supplementary Materials.

Melting points (mp) were determined using a Stuart Scientific SMP3 apparatus (Staffordshire, UK) and are uncorrected. Infrared (IR) spectra were recorded on a Bruker FT-IR spectrophotometer (Vector 22 model; Bruker, Rheinstetten, Germany) using KBr disks, and the wavenumbers are reported in cm^−1^. ^1^H–NMR spectra were recorded on Bruker Avance-400 and Bruker 200 spectrometers (Bruker, Ettlingen, Germany) in DMSO-*d*_6_ or CDCl_3_. ^13^C–NMR spectra were obtained in DMSO-*d*_6_ or CDCl_3_ at 100 and 50 MHz. Chemical shifts (δ) are reported in parts per million (ppm) downfield relative to tetramethylsilane (TMS) as the internal standard, and coupling constants (*J*) are expressed in Hertz (Hz). The data for the ^1^H–NMR spectra are reported as follows: s (singlet), br s (broad singlet), d (doublet), t (triplet), and m (multiplet). Bidimensional NMR techniques and distortion-less enhancement by polarization transfer (DEPT) were used for signal assignment. Silica gel Merck 60 (70–230 mesh) was used for preparative column chromatography and aluminum foil 60F_254_ (Merck) was used for analytical thin-layer chromatography (TLC).

#### 2.1.2. Solvent-Free Synthesis of Paracetamol Analogues 

The solvent-free, silica-assisted grinding approach was selected to align with green chemistry principles and enhance reaction efficiency. Preliminary solution-phase trials resulted in longer reaction times and lower isolated yields, whereas the mechanochemical protocol provided faster conversion and improved reproducibility while significantly reducing solvent consumption.

**Figure 1:**
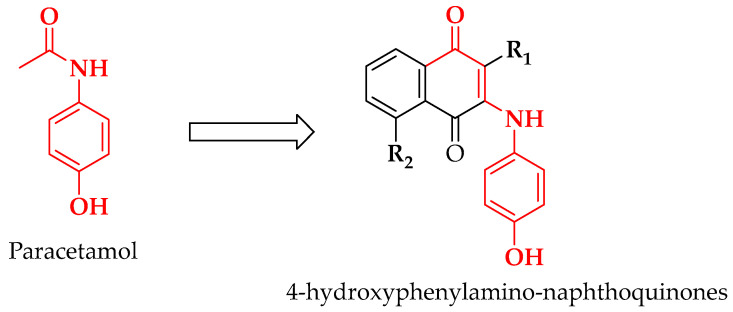
Design strategy for paracetamol-inspired 4-hydroxyphenylamino-naphthoquinones integrating key structural features of paracetamol with a redox-active naphthoquinone core.

Guided by the design strategy outlined in Figure 1, electronically and sterically distinct 1,4-naphthoquinones were selected as Michael acceptors and combined with 4-hydroxyaniline to generate paracetamol-inspired 4-hydroxyphenylamino-naphthoquinones.

A mixture of quinones—namely, 1,4-naphthoquinone **1**, 2,3-dichloro-1,4-naphthoquinone **2**, 5-hydroxy-1,4-naphthoquinone **3**, or 5-nitro-1,4-naphthoquinone **4** (1.0 mmol)—and the corresponding 4-hydroxyaniline (1.0 mmol) were supported on silica gel (0.4 g) and thoroughly homogenized in an agate mortar. The loading (~0.4 g per mmol) was experimentally optimized; lower amounts resulted in incomplete conversion, whereas higher amounts did not improve yields. Equimolar stoichiometry was adopted for reproducibility, and under these mechanochemical conditions, exclusive monosubstitution at the quinone core was consistently observed, with no detectable over-addition products. Reaction progress was monitored every 15 min by TLC, and completion was defined as the complete disappearance of the starting quinone and the appearance of a stable predominant product band under UV detection.

The crude mixture was purified by short silica gel filtration and eluted with petroleum ether/ethyl acetate to afford the isolated products.

**Figure 2:**
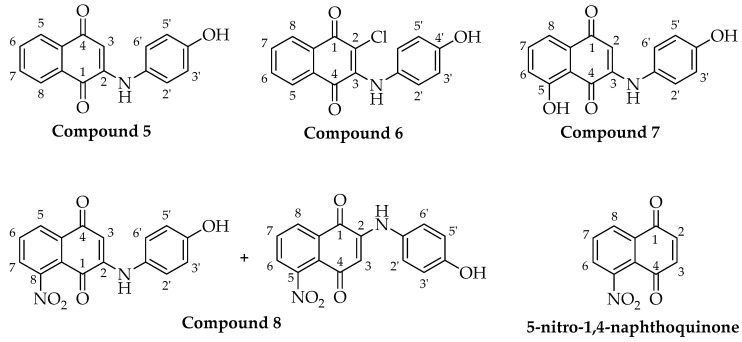
Compounds **5**–**7**, the two regioisomers of compound **8**, and 5-nitro-1,4-naphthoquinone.

The spectroscopic characterization of the compounds shown in Figure 2 is detailed below:

2-((4-hydroxyphenyl)amino)-1,4-naphthoquinone **5**. (89%), brown solid, mp: 255.1–255.5 °C. IR (KBr) ν_max_ cm^−1^: 3301 (OH); 3219 (NH); 1670 (C=O); 1622 (C=O). ^1^H NMR (400 MHz, DMSO–*d*_6_) δ: 9.57 (s, 1H, OH), 9.04 (s, 1H, NH), 8.03 (d, *J* = 7.6 Hz, 1H, H−8), 7.92 (d, *J* = 7.5 Hz, 1H, H−5), 7.83 (t, *J* = 7.4 Hz, 1H, H−7), 7.75 (t, *J* = 7.5 Hz, 1H, H−6), 7.16 (d, *J* = 8.4 Hz, 2H, H−2′ + H−3′), 6.83 (d, *J* = 8.3 Hz, 2H, H− 5′ + H−6′), 5.87 (s, 1H, H−3). ^13^C NMR (100 MHz, DMSO–*d*_6_) δ: 182.12, 181.72, 155.35, 147.06, 134.86, 132.84, 132.41, 130.44, 128.98, 126.03, 125.79 (2C), 125.25, 115.82 (2C), 100.78. (Figures S1 and S2, Supplementary Material).

2-cloro-3-((4-hydroxyphenyl)amino)-1,4-naphthoquinone **6.** (78.7%), purple solid, mp: 235.1–236.4 °C. IR (KBr) ν_max_ cm^−1^: 3295 (NH); 3220 (OH); 1671 (C=O); 1595(C=O) 718 (C−Cl). ^1^H NMR (400 MHz, DMSO–*d*_6_) δ: 9.42 (s, 1H, OH), 9.13 (s, 1H, NH), 8.06–7.96 (m, 2H, H−5 + H−8), 7.84 (t, *J* = 7.5 Hz, 1H, H−6), 7.77 (t, *J* = 7.6 Hz, 1H, H−7), 6.97 (d, *J* = 8.2 Hz, 2H, H−2′ + H−3′), 6.71 (d, *J* = 8.2 Hz, 2H, H−5′ + H−6′). ^13^C NMR (100 MHz, DMSO–*d*_6_) δ: 180.19, 176.38, 155.02, 143.45, 134.86, 132.95, 132.17, 130.07, 130.00, 126.48, 126.31 (2C), 126.01, 114.52 (2C), 111.58. (Figures S3 and S4, Supplementary Material).

3-((4-hydroxyphenyl)amino)-5-hydroxy-1,4-naphthoquinone **7.** (50%), brown solid, mp: 243–245 °C. IR (KBr) ν_max_ cm^−1^: 3383 (OH); 3339 (OH); 3286 (NH); 1718 (C=O) 1630 (C=O). ^1^H NMR (400 MHz, DMSO–*d*_6_) δ: 11.43 (brs, 1H, 5−OH), 9.53 (s, 1H, 4′−OH), 9.0 (s, 1H, –NH), 7.71 (t, 1H, *J* = 7.9 Hz, H-7), 7.44 (d, 1H, *J* = 7.4 Hz, H-8), 7.22 (d, 1H, *J* = 8.4 Hz, H-6), 7.15 (d, 2H, *J* = 8.5 Hz, H-2′ + H-6′), 6.84 (d, 2H, *J* = 8.4 Hz, H-3′ + H-5′), 5.81 (s, 1H, H-2). ^13^C NMR (100 MHz, DMSO–*d*_6_) δ: 185.72, 181.34, 160.45, 155.38, 147.00, 137.45, 133.14, 128.79, 125.79 (2C), 121.89, 117.48, 115.79 (2C), 114.21, 101.08. (Figures S5–S7, Supplementary Material).

2-((4-hydroxyphenyl)amino)-8-nitro-1,4-naphthoquinone + 2-((4-hydroxyphenyl)amino)-5-nitro-1,4-naphthoquinone **8.** (89.7%), purple solid, mp: 165.1–166.7 °C. IR (KBr) ν_max_ cm^−1^: 3656 (OH); 2922 (NH); 1682 (C=O) 1668 (C=O); 1512 (NO_2_). ^1^H NMR (400 MHz, DMSO–*d*_6_) δ: 9.61 (s, 2H, –NH), 9.33 (s, 1H, –OH), 9.26 (s, 1H, –OH), 8.23 (d, *J* = 8.2 Hz, 1H), 8.14 (dd, *J* = 6.9, 2.0 Hz, 2H), 8.04 (dd, *J* = 8.1, 2.3 Hz, 2H), 8.00 (d, *J* = 7.9 Hz, 1H), 7.91 (t, *J* = 7.9 Hz, 1H), 7.15 (dd, *J* = 8.7, 5.9 Hz, 4H), 6.83 (d, *J* = 8.7 Hz, 3H), 5.89 (s, 1H, H–3), 5.82 (s, 1H, H–3). ^13^C NMR (100 MHz, DMSO–*d*_6_) δ 179.89, 179.84, 178.96, 178.67, 155.59 (2C), 147.73, 147.24, 147.08, 136.10 (2C), 133.82, 133.57, 131.77, 128.64, 128.45, 128.08, 127.74 (2C), 126.04 (2C), 125.83 (4C), 120.97, 115.88 (4C), 100.89, 100.52. HMBC analysis enabled reliable regioisomeric assignment. The minor component (δH 5.82 ppm) exhibited long-range correlations consistent with the 2-((4-hydroxyphenyl)amino)-8-nitro-1,4-naphthoquinone structure, including correlations from H-3 and the diagnostic aromatic proton H-5 (δH 8.23 ppm) to the quinone carbonyl carbons at δC 179.89 and 179.84 ppm. In contrast, the major component (δH 5.89 ppm) displayed HMBC correlations to the carbonyl carbons at δC 178.96 and 178.67 ppm, consistent with substitution at C-5. Accordingly, the major regioisomer (~66%) was assigned as the 2-((4-hydroxyphenyl)amino)-5-nitro-1,4-naphthoquinone derivative (Figures S8–S11, Supplementary Material). 

5-Nitro-1,4-naphthoquinone, the precursor used for the synthesis of compound **8**, was prepared by nitration of 1,4-naphthoquinone. To this end, a mixture of 1,4-naphthoquinone (1.0 mmol) in water (1.68 mL), nitric acid (HNO_3_, 65%, 3.4 mL), and sulfuric acid (H_2_SO_4_, 97%, 19 mL) was prepared. The reaction mixture was stirred at 0–5 °C in an ice/acetone bath for 7 h. The reaction was quenched by the addition of ice. The resulting mixture was filtered, and the residue was dissolved in ethyl acetate. The organic phase was washed with NaHCO_3_ solution and distilled water (3 × 20 mL), dried over anhydrous magnesium sulfate, filtered, and concentrated under reduced pressure. Finally, the crude product was purified by column chromatography on silica gel using a petroleum ether/ethyl acetate mixture as the mobile phase for elution.

5-nitro-1,4-naphthoquinone. (78%), yellow solid, mp: 165.1–166.7 °C. ^1^H NMR (200 MHz, CDCl_3_) δ: 8.19 (m, *J* = 7.4, 1.5 Hz, 1H, H−8), 7.98–7.79 (m, 2H, H−6 + H−7), 7.09–6.92 (m, 2H, H−2 + H−3). ^13^C NMR (50 MHz, CDCl_3_) δ: 181.41, 180.42, 146.93, 137.87, 137.23, 134.07, 131.46, 127.67, 127.55, 121.42. (Figures S12 and S13, Supplementary Material).

### 2.2. In Silico Studies

#### 2.2.1. Prediction of Biological Activity Based on Spectra

The prediction of the biological activity of the compounds **5**–**8** was carried out by spectral analysis using the PASS server, freely available at https://www.way2drug.com/PassOnline (accessed 6 September 2025). The activity spectrum of a molecule provides a list of biological activities along with the probabilities of the compound being active (*Pa*) or inactive (*Pi*), with values ranging from 0 to 1 [27]. In this study, PASS predictions were focused on pharmacologically relevant endpoints, including antinociceptive activity, analgesic stimulant effects, vanilloid receptor agonism, anti-inflammatory activity, cyclooxygenase inhibition (COX-1 and COX-3), and antipyretic activity.

#### 2.2.2. Chemical Reactivity Based on Density Functional Theory (DFT)

The chemical reactivity descriptors, namely, chemical potential (µ) and chemical hardness (η), were calculated using the so-called conceptual DFT [28]. The electronic and structural properties of the paracetamol-analog quinonoid compounds were initially treated using the Universal Force Field (UFF) [29] and subsequently re-optimized at the B3LYP level of theory in conjunction with the 6-311G(d,p) basis set using Gaussian 16 [30,31,32,33,34]. All optimized structures were confirmed to correspond to the true energy minima by the absence of imaginary vibrational frequencies.

To account for implicit solvent effects, water was modeled using the Polarizable Continuum Model [35]. The chemical reactivity indices were calculated according to Koopmans’ theorem using the following equations:

µ = −½ (*I* + *A*)

η = ½ (*I* − *A*)

where *I* and *A* correspond to the ionization energy and electron affinity, respectively. According to Koopmans’ approximation, the ionization energy (*I*) was obtained as the negative of the energy of the highest occupied molecular orbital (HOMO), whereas the electron affinity (*A*) was estimated as the negative of the energy of the lowest unoccupied molecular orbital (LUMO).

#### 2.2.3. Molecular Docking 

Compounds **5**–**7**, the two regioisomers of compound **8**, PCM, and AM404, were docked onto protein targets, including cyclooxygenase 2 (COX-2, PDB code 5IKR) [36], transient receptor potential vanilloid 1 (TRPV1, PDB code 3J5P) [37], and cannabinoid receptor (CB1, PDB code 5U09) [38]. Docking analyses involving TRPV1 and CB1 were performed for exploratory purposes and are reported in the Supplementary Materials (Table S2 and Figures S14 and S15). 

Molecular docking was performed using AutoDock (v 4.2.1, Scripps Research Institute, San Diego, CA, USA), AutoDock Vina (v 1.0.2 Scripps Research Institute, San Diego, CA, USA) [39], and the AutoDockTools package v 1.5.7 [40], following previously described protocols [41]. Validation was performed by re-docking the co-crystallized ligands into their corresponding binding sites, yielding RMSD values < 2.0 Å, confirming the reliability of the docking protocol. All other parameters were set to the AutoDock Vina default. Docking simulations were repeated 20 times, with the exhaustiveness parameter set to 100. The best binding energy values (kcal·mol^−1^) were selected for evaluation. Three-dimensional docking results were visualized using Discovery Studio 3.1 (Accelrys, San Diego, CA, USA).

#### 2.2.4. ADMET Prediction

The pkCSM online tool (http://biosig.unimelb.edu.au/pkcsm/prediction, accessed on 23 August 2025) [42], was utilized to predict absorption, distribution, metabolism, excretion and toxicity (ADMET) of compounds **5**–**7**, and the two regioisomers of compound **8**.

### 2.3. Pharmacological Evaluation

#### 2.3.1. Animals

The experiments in this study were conducted in accordance with the guidelines of the American Veterinary Medical Association (AVMA) and were approved by the Ethics Committee of the Pharmacy and Biochemistry Faculty at the National University of Trujillo (COD N° 012-2025/CE). Two hundred and sixty-four male *Rattus norvegicus* var. Holtzman (8–10 weeks old and 170–200 g) were used. Animals were housed at 22–25 °C under a 12 h light/dark cycle with ad libitum access to food and water.

#### 2.3.2. Experimental Design 

Animals were randomly assigned to control and experimental groups (n = 5 per group). The animals in the control group received the vehicle (Tween 80), whereas those in the reference group were treated with PCM in the antinociceptive and antipyretic models, naproxen (NPX) in the anti-inflammatory model, and the test groups (compounds **5**–**8**), as described in Table 1. The selected doses (12.5 and 25 mg/kg) were chosen based on literature reports demonstrating effective antinociceptive and anti-inflammatory activity for PCM and NPX within the 10–30 mg/kg range in comparable rodent models [43,44]. These doses fall within the pharmacologically active but non-toxic range reported for acute testing. 

#### 2.3.3. Antinociceptive Assay

The analgesic (antinociceptive) activity of the compounds was evaluated using Eddy’s hot plate and acetic acid-induced writhing assays [18].

##### Eddy’s Hot Plate

The animals were placed on a hot plate analgesiometer maintained at 55−56 °C, a temperature selected according to established supraspinal nociception protocols to elicit a reliable and reproducible thermal pain response while preventing excessive tissue injury. The time to paw licking or jumping response was recorded, and a cutoff time of 15 s was applied to avoid tissue damage. The analgesic potential was evaluated based on the latency of response before and 60, 120, 180, 240, and 300 min post-administration. The extended 300 min observation period was designed to capture both early and sustained analgesic effects and to enable area-under-the-curve (AUC) analysis. The treatment received by the animals allocated to the different experimental groups was in accordance with the study design, as described in Table 1.

##### Acetic Acid-Induced Writhing Effect

The injection of an irritant into the peritoneal cavity is known to induce pain and characteristic writhing in rats. A 1 mL dose of 0.5% acetic acid solution was injected intraperitoneally (i.p.) into the experimental animals following pre-treatment with the test and standard drugs, as per the study design (Table 1). The animals were placed individually in glass beakers, and the number of writhes was recorded for 20 min after the challenge. 

#### 2.3.4. Anti-Inflammatory Assay

Anti-inflammatory activity was evaluated using a carrageenan-induced paw edema model [45].

##### Carrageenan-Induced Paw Edema Assay

The carrageenan-induced rat paw edema assay was employed to assess anti-inflammatory activity. Carrageenan (1%, 0.05 mL, subcutaneous) was selected according to standardized acute inflammation protocols that reliably induce a biphasic inflammatory response characterized by early mediator release, followed by a prostaglandin-dependent phase. The selected dose and sampling times were chosen to capture both phases of the inflammatory response. The animals were fasted overnight with free access to water. Prior to carrageenan administration, the animals were allocated to different experimental groups and treated according to the study design described in Table 1. After 60 min, 0.05 mL of 1% carrageenan solution was injected (s.c.) into the plantar region of the left hind paw. The right paw was used as a control. Changes in paw volume were measured plethysmographically at 0, 30, 60, 90, and 120 min after carrageenan injection.

#### 2.3.5. Antipyretic Assay [46]

The experiments were performed during the light phase of the circadian cycle in rats. Body temperature (Tb) was measured by gently inserting a digital thermometer 4 cm into the rectum for a maximum of 1 min. During temperature measurements, each animal was handled manually and gently without removal from its home cage. This procedure was performed at least twice during the two days preceding the experiments to minimize rectal temperature changes induced by handling. On the day of the experiment, the Tb of each animal was allowed to stabilize for 1 h, and the baseline temperature was recorded three times at 30 min intervals. Only animals exhibiting stable Tb values within the range of 36.8–37.8 °C were included in the study to reduce inter-animal variability and ensure consistent induction of pyrexia. Each animal was used only once. Animals were allocated to different experimental groups according to the study design described in Table 1. After 30 min, lipopolysaccharide (LPS, 100 µg/kg, i.p.) dissolved in pyrogen-free sterile saline was administered. This dose was selected based on established rodent fever models that reliably induce a cytokine-mediated febrile response. Body temperature was recorded at 1, 2, 3, 4, 5, and 6 h after LPS administration to capture the temporal profile of the febrile response.

### 2.4. Statistical Analysis

All data from the in vivo experimental studies were subjected to statistical analysis. Data normality was assessed using the Shapiro–Wilk test. The results are presented as the mean ± standard error of the mean (SEM). Differences in mean values were analyzed using two-way ANOVA, followed by Tukey’s multiple comparisons test using GraphPad Prism 8.0.2 software (San Diego, CA, USA). *p* < 0.05 value was considered statistically significant.

## 3. Results

### 3.1. Solvent-Free Synthesis 

The condensation of 1,4-naphthoquinone with 4-hydroxyaniline was first examined as a model reaction under solvent-free conditions. In the absence of silica gel, the Michael adduct formed slowly, reaching completion after 15 h, with an isolated yield of 65 %. In contrast, adsorption of equimolar reactants (1 mmol each) onto silica gel (0.4 g) markedly accelerated the reaction, affording the desired product within 30 min with an 89% yield (Table 2). The silica gel was recovered by filtration, washed with acetone, dried at 100 °C, and reused. These optimized conditions were subsequently applied to substituted naphthoquinones (**2**–**4**), providing the corresponding 4-hydroxyphenylamino-naphthoquinones in yields ranging from 50.0 to 89.7%, with reaction times ranging from 1 to 8 h. In most cases, a single product was obtained. However, compound **4** afforded an inseparable regioisomeric mixture (compound **8**). This mixture was evaluated as a structurally defined system, with a major-to-minor ratio of approximately 66:34, as determined by quantitative integration of diagnostic H-3 signals in the ^1^H NMR spectrum. The major regioisomer was assigned as the 5-nitro derivative based on 2D NMR (HMQC/HMBC) correlations.

### 3.2. PASS-Based Prediction of Biological Activity

The potential biological activities of the synthesized compounds were evaluated using the PASS online server and the PharmaExpert platform. The predicted activities of compounds **5**–**8** are summarized in Table 3, with paracetamol (PCM) included as the reference compound. The PASS outputs are reported as the probability of activity (Pa) and the probability of inactivity (Pi). Because compound **8** was experimentally obtained as a regioisomeric mixture, PASS predictions were also evaluated for the individual regioisomers. However, the PASS server generated essentially identical Pa/Pi values for both structures, reflecting the use of global structural descriptors in the algorithm. Therefore, a single set of PASS predictions is presented for compound **8** in Table 3. 

For compound **5**, PASS analysis predicted antinociceptive activity with a Pa value of 0.345, with the corresponding Pi value being lower than the Pa value. No relevant Pa values associated with antinociceptive activity were obtained for compound **8**. Predicted vanilloid receptor 1 (TRPV1) agonist activity was observed for all compounds (**5**–**8**), with Pa values ranging from approximately 0.212 to 0.396 and low Pi values. Additionally, all evaluated compounds were predicted to exhibit anti-inflammatory activity, with Pa values ranging from approximately 0.239 to 0.427. The predicted inhibition of cyclooxygenase isoforms, including cyclooxygenase-1 and cyclooxygenase-3, was also observed for several compounds with moderate Pa values. 

Predicted antipyretic activity was identified for compounds **5** and **7**, with Pa values comparable to those calculated for PCM.

### 3.3. Density Functional Theory (DFT) Calculations

To gain insight into the electronic properties of the synthesized compounds, geometry optimizations were performed using density functional theory (DFT). The optimized structures of compounds **5**–**7** and the two regioisomers of compound **8** are shown in Figure S16 (Supplementary Material). Because compound **8** was experimentally obtained as an inseparable regioisomeric mixture, DFT calculations were performed independently for each regioisomer to evaluate the potential influence of the nitro substitution pattern on the electronic properties of the scaffold. 

Based on the optimized geometries, key quantum chemical descriptors related to electronic stability and reactivity were calculated. The resulting parameters for compounds **5**–**7**, the two regioisomers of compound **8**, and the reference drug paracetamol (PCM) are summarized in Table 4 and were obtained at the B3LYP/6-311G(d,p) level of theory. The ionization potential (I), electron affinity (A), chemical potential (µ), and chemical hardness (η) provide insights into the electronic stability and reactivity of the studied compounds.

Compounds **5**–**7** showed closely clustered ionization potentials (133.9–137.3 kcal·mol^−1^), comparable to PCM (134.8 kcal·mol^−1^). For compound **8**, DFT descriptors were calculated for each regioisomer separately; the 5-nitro (major) and 8-nitro (minor) isomers displayed slightly higher I values (137.0 and 136.8 kcal·mol^−1^, respectively), indicating a broadly similar electron-donation propensity across the series

In contrast, the electron affinities for compounds **5**–**8** (including both regioisomers of compound **8**) were substantially higher than those for PCM, consistent with a more stabilized LUMO in the naphthoquinone derivatives. The calculated A values ranged from 69.7 to 79.0 kcal·mol^−1^ for compounds **5**–**7** and the two regioisomers of compound **8**, whereas PCM showed a markedly lower value (12.2 kcal·mol^−1^), indicating a significantly greater electron-accepting capacity of the naphthoquinone scaffold, consistent with the intrinsically electron-deficient nature of quinone systems. This behavior reflects the enhanced stabilization of the LUMO in compounds **5**–**8** relative to PCM.

The calculated chemical potential (µ) values for compounds **5**–**7** and the two regioisomers of compound **8** ranged from −101.8 to −107.9 kcal·mol^−1^, which were more negative than that of PCM (−73.5 kcal·mol^−1^). This trend suggests a stronger tendency of these compounds to attract electron density, consistent with their higher electron affinity and increased electrophilic character. Regarding chemical hardness (η), compounds **5**–**7** and the regioisomers of compound **8** displayed substantially lower values (28.9–32.1 kcal·mol^−1^) than PCM (61.3 kcal·mol^−1^). The reduced hardness indicates a smaller HOMO–LUMO energy gap and, therefore, a higher electronic polarizability and reactivity of the naphthoquinone derivatives relative to PCM. 

Notably, the two regioisomers of compound **8** showed highly similar electronic descriptors, indicating that the position of the nitro substituent exerts only a minor influence on the overall electronic distribution of the naphthoquinone scaffold.

### 3.4. Docking Molecular 

To explore the potential molecular interactions associated with inflammation-related pain, molecular docking analyses were performed for compounds **5**–**7**, the two regioisomers of compound **8**, PCM, and AM404 using the COX-2 enzyme (PDB ID: 5IKR). Because compound **8** was evaluated in vivo as a ~2:1 regioisomeric mixture, docking simulations were run separately for both regioisomers. These results are reported as qualitative structural descriptors and cannot be directly correlated with the biological activity of the experimental mixture.

As shown in Table 5, all compounds exhibited favorable predicted binding affinities toward COX-2. Compounds **5** and the minor regioisomer of compound **8** showed the lowest binding energies (−9.9 kcal/mol), whereas compounds **6**, **7**, and the major regioisomer of compound **8** displayed values of −9.7 kcal/mol. In contrast, PCM showed a weaker binding energy (−6.1 kcal/mol), whereas AM404 exhibited an intermediate value (−8.2 kcal/mol). These docking results provide structural hypotheses regarding ligand–target complementarity and are presented for exploratory purposes only. They do not constitute evidence of functional target engagement or mechanistic equivalence with paracetamol or its metabolites.

**Figure 3:**
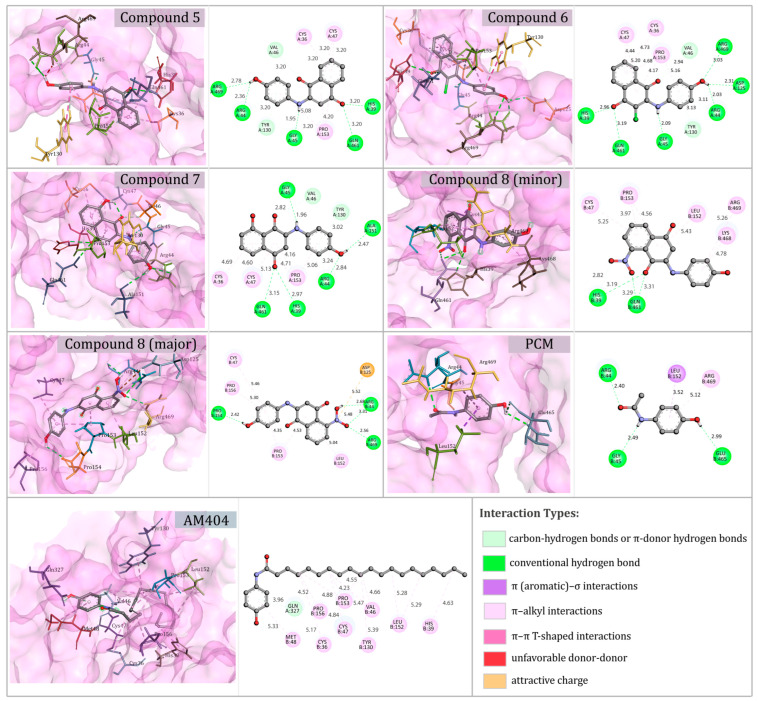
Representative docking poses and interaction profiles of compounds **5**–**7**, the two regioisomers of compound **8**, paracetamol (PCM), and *N*-arachidonoylphenolamine (AM404) within the COX-2 enzyme-binding site. The left panels show the overall receptor structure with the ligand-binding region highlighted, whereas the middle panels depict a magnified view of the binding pocket illustrating the ligand orientation and key interacting residues. Hydrogen atoms are omitted in some cases for the sake of clarity.

The docking interactions shown in Figure 3 depict the predicted binding modes of compounds **5**–**7**, the two regioisomers of compound **8** (minor and major), and the reference ligands, PCM and AM404, within the catalytic cavity of the COX-2 enzyme. In all the analyzed systems, the ligand–protein complexes were stabilized by combinations of conventional hydrogen bonds, carbon–hydrogen or π-donor hydrogen bonds, π–alkyl interactions, and π–π or π–σ interactions. In some cases, these interactions were accompanied by less favorable donor–donor contacts.

All ligands occupied a similar region within the active site and adopted comparable orientations within the catalytic pocket. The synthesized compounds formed multiple interactions with residues such as Arg44, Arg469, His39, Gln461, Gly45, Tyr130, and neighboring residues within the catalytic channel. Conventional hydrogen bonds and carbon–hydrogen or π-donor hydrogen bonds were frequently observed, generally with interaction distances below 3.5 Å. Additional π–alkyl, π(aromatic)–σ, and π–π T-shaped interactions were also detected. The two regioisomers of compound **8** displayed interaction profiles comparable to those observed for compounds **5**–**7** within the COX-2 binding cavity. In contrast, PCM showed fewer hydrogen-bond interactions and a reduced number of π-related contacts within the enzyme-binding pocket. AM404, in turn, exhibited a broader interaction profile, including a conventional hydrogen bond with Glu513 and Arg491. π-related interactions (π–π and π–alkyl) were also observed, and its aliphatic chain extended toward a hydrophobic region of the catalytic channel, contributing to additional van der Waals interactions. Overall, these features suggest a more extensive interaction network for AM404 than for PCM within the COX-2 binding site.

### 3.5. In Silico ADMET Prediction of Compounds ***5***–***7*** and the Two Regioisomers of Compound ***8***

The in silico ADMET properties of compounds **5**–**7** and the two regioisomers of compound **8** were estimated using the pkCSM platform for an initial computational screening of pharmacokinetic and toxicity-related parameters. These predictions provide theoretical estimates based on quantitative structure–property relationships and do not constitute experimental evidence of pharmacokinetic or safety advantages over reference drugs. Because compound **8** was experimentally evaluated as an approximately 2:1 regioisomeric mixture, ADMET predictions were generated independently for each regioisomer and should therefore be interpreted as qualitative indicators rather than as a direct representation of the pharmacokinetic behavior of the tested mixture. Table 6 summarizes the in silico ADMET properties of compounds **5**–**7**, the two regioisomers of compound **8**, and PCM as a reference compound.

Absorption: Compounds **5**–**7** were predicted to exhibit favorable intestinal absorption profiles, with Caco-2 permeability values above 1.0 (1.018–1.038), comparable to that predicted for PCM (1.231). In contrast, both regioisomers of compound **8** showed lower predicted Caco-2 permeability values (0.546 and 0.516), suggesting reduced membrane permeability relative to compounds **5**–**7**. Predicted intestinal absorption (IA) values were high for all compounds (89.0–93.9%), indicating potential for oral absorption. The predicted skin permeability (SP) values were negative and within a similar range for all compounds (−2.749 to −3.611), comparable to PCM (−2.832), suggesting limited transdermal penetration.

Distribution: The predicted steady-state volume of distribution (VDss) values for compounds **5**–**7** were negative and comparable to that predicted for PCM, suggesting moderate systemic distribution. In contrast, the two regioisomers of compound **8** showed slightly positive VDss values (0.050 and 0.084), indicating a somewhat different predicted distribution behavior within the series. Predicted blood–brain barrier (BBB) permeability values were negative for all compounds, including the regioisomers of compound **8**, suggesting limited BBB permeability. Predicted CNS permeability values ranged from −2.037 to −2.227 for compounds **5**–**7** and from −2.217 to −2.215 for the two regioisomers of compound **8**, remaining close to the threshold commonly associated with restricted central nervous system exposure in the pkCSM model. Therefore, these predictions are consistent with limited brain distribution; however, experimental pharmacokinetic studies are required to confirm this behavior.

Metabolism: Regarding predicted metabolic interactions, compounds **5** and **7** were predicted not to inhibit CYP2D6 or CYP3A4. Compound **6** was predicted to inhibit CYP3A4 but not CYP2D6. Among the two regioisomers of compound **8**, the minor regioisomer was predicted not to inhibit either enzyme, whereas the major regioisomer showed predicted inhibition of CYP3A4 in the pkCSM model. PCM was similarly predicted not to inhibit either enzyme. These results suggest possible variability in predicted CYP-mediated metabolic interactions within the series; however, experimental metabolic studies are warranted to validate these computational predictions.

Excretion: Predicted total clearance (TC) values for compounds **5**–**7** ranged from 0.049 to 0.137, whereas the two regioisomers of compound **8** showed lower predicted clearance values (0.042 and 0.040). These values were substantially lower than those predicted for the PCM (0.487). Within the pkCSM model, this pattern may suggest slower predicted elimination for the naphthoquinone derivatives; however, these estimates should be interpreted cautiously because they are derived exclusively from computational predictions and do not represent experimentally measured pharmacokinetic parameters. 

Toxicity: All compounds were predicted to exhibit acute oral toxicity (LD_50_) values in rats within a comparable range. Compounds **5**–**7** exhibited predicted LD_50_ values between 2.241 and 2.305, similar to PCM (2.166). The two regioisomers of compound **8** showed slightly higher predicted LD_50_ values (2.770 and 2.813), suggesting no major increase in predicted acute toxicity within the model. Predicted chronic oral toxicity (LOAEL) values ranged from 1.831 to 2.086 across the series, remaining within the same general order of magnitude as PCM. These predictions indicate no major differences in estimated toxicity descriptors within the computational model; however, experimental safety and toxicological studies are required to confirm these predictions.

### 3.6. Central Antinociceptive Effect of Compounds ***5***–***8***

**Figure 4:**
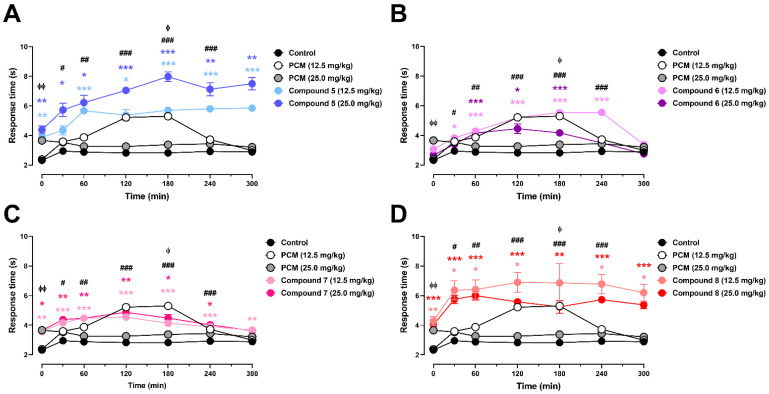
Time-course of central antinociceptive effects of paracetamol-inspired analogs (compounds **5**–**8**) in the hot plate assay. Compounds **5** (**A**), **6** (**B**), **7** (**C**), and **8** (**D**) were evaluated over a 300 min observation period. Data are presented as the mean ± SEM (n = 4–8 animals per group). Statistical analysis was performed using two-way ANOVA, followed by Tukey’s multiple comparisons test. Statistical significance versus the control is indicated as * *p* < 0.05; ** *p* < 0.01; *** *p* < 0.001 for the test compounds; # *p* < 0.05; ## *p* < 0.01; ### *p* < 0.001 for PCM (12.5 mg/kg), and ^ϕ^ *p* < 0.05; ^ϕϕ^ *p* < 0.01 for PCM (25.0 mg/kg). The colors of the asterisks and symbols correspond to the respective experimental groups. Compound **8** was experimentally evaluated as an inseparable regioisomeric mixture (~2:1 major:minor).

To assess whether the paracetamol-inspired analogs exerted centrally mediated antinociceptive effects, nociceptive response latencies were measured in the hot plate test over a 300 min observation period (Figure 4). 

As shown in Figure 4A, compound **5** produced a rapid onset and a robust and sustained increase in response latency. Baseline latencies were comparable between the control group (2.32 ± 0.19 s) and PCM at 12.5 mg/kg (2.38 ± 0.20 s), whereas compound **5** exhibited significantly higher baseline values at both 12.5 mg/kg (3.88 ± 0.03 s, *p* < 0.01 vs. control) and 25 mg/kg (4.38 ± 0.28 s, *p* < 0.01 vs. control). At 30 min, latency increased markedly, particularly at 25 mg/kg (5.73 ± 0.46 s, *p* < 0.05 vs. control). This effect intensified at 60 min (5.68 ± 0.10 s at 12.5 mg/kg, *p* < 0.001; 6.23 ± 0.49 s at 25 mg/kg, *p* < 0.05 vs. control) and remained significant throughout the later phases of the experiment (120–300 min). Peak latencies were recorded between 180 and 300 min at 25 mg/kg (7.98 ± 0.33 s and 7.50 ± 0.42 s, respectively; *p* < 0.001 vs. control), clearly exceeding the corresponding control values (2.83–2.89 s).

In Figure 4B, compound **6** elicited a moderate but statistically significant central antinociceptive effect, characterized by a delayed maximum and partial decay at the end of the observation period. Baseline latencies were slightly higher than those in the control group at both doses (3.08 ± 0.25 s at 12.5 mg/kg and 2.68 ± 0.17 s at 25 mg/kg). At 30 min, latency increased to 3.83 ± 0.14 s at 12.5 mg/kg, whereas at 60 min, both doses produced significant elevations (4.29 ± 0.07 s at 12.5 mg/kg and 4.16 ± 0.11 s at 25 mg/kg; *p* < 0.01 vs. control). Maximal responses were observed between 180 and 240 min, with latencies reaching 5.53 ± 0.11 s and 5.54 ± 0.10 s at 12.5 mg/kg (*p* < 0.001 vs. control at both time points). Notably, the 25 mg/kg dose yielded a comparatively less pronounced increase in the mid-to-late phase. By 300 min, the latencies declined toward baseline values, indicating a time-limited central antinociceptive action under these conditions.

As shown in Figure 4C, compound **7** induced an early but transient increase in nociceptive latency. Baseline values were higher than those of the control at both doses (3.58 ± 0.09 s at 12.5 mg/kg and 3.65 ± 0.19 s at 25 mg/kg). At 30 min, significant increases were observed in the 12.5 and 25 mg/kg groups (4.18 ± 0.09 s and 4.38 ± 0.15 s; *p* < 0.001 and *p* < 0.01 vs. control, respectively). The peak effects occurred between 60 and 120 min, with latencies of 4.48 ± 0.06 s (12.5 mg/kg) and 4.88 ± 0.21 s (25 mg/kg), both significantly different from the control (*p* < 0.001). Thereafter, the latency values progressively declined, approaching control levels at later time points, consistent with a short-duration central antinociceptive profile.

Finally, compound **8** showed the most pronounced and persistent elevation in response latency among the evaluated analogs (Figure 4D). Baseline latencies were markedly increased relative to the control at both doses (4.28 ± 0.30 s at 12.5 mg/kg and 4.03 ± 0.21 s at 25 mg/kg). At 30 min, compound **8** induced a strong increase in latency, particularly at 12.5 mg/kg (6.36 ± 0.65 s, *p* < 0.05 vs. control), and this effect remained significant at 60 min (6.40 ± 0.66 s at 12.5 mg/kg, *p* < 0.05; 6.00 ± 0.30 s at 25 mg/kg, *p* < 0.001). Elevated latencies persisted throughout the experiment, with maximal responses observed between 120 and 180 min (up to 6.90 ± 0.67 s; *p* < 0.05 vs. control). Importantly, the effect was still evident at 300 min (6.20 ± 0.57 s and 5.38 ± 0.26 s for 12.5 and 25 mg/kg, respectively).

### 3.7. Peripheral Antinociceptive Effect of Compounds ***5***–***8***

**Figure 5:**
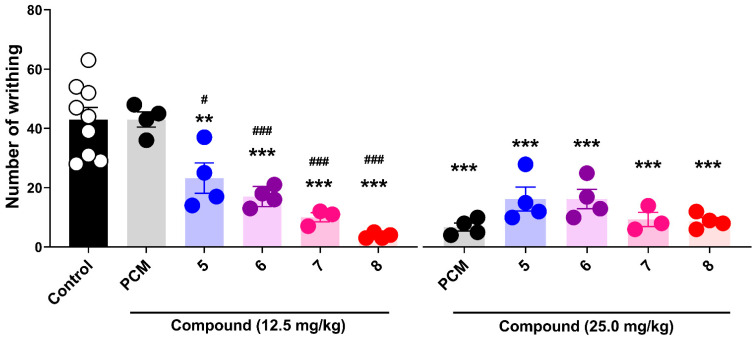
Peripheral analgesic activity of paracetamol-inspired analogs in the acetic acid-induced writhing test. Peripheral nociception was induced by intraperitoneal administration of acetic acid, and the number of writhes was recorded for 20 min, starting 5 min after the challenge. The animals received PCM (paracetamol, 12.5 and 25 mg/kg) or compounds **5**–**8** at the same doses. No statistically significant dose-dependent differences were detected within the tested range for the synthesized compounds. Data are presented as mean ± SEM (n = 3–9 animals per group). Statistical analysis was performed using two-way ANOVA followed by Tukey’s multiple comparisons test. Statistical significance versus the control is indicated as ** *p* < 0.01 and *** *p* < 0.001; while significance vs. the PCM group is indicated as # *p* < 0.05 and ### *p* < 0.001.

Figure 5 illustrates the effects of paracetamol-inspired analogs (compounds **5**–**8**) and PCM on acetic acid-induced writhing in rats at doses of 12.5 and 25 mg/kg. The control group exhibited a mean of 43.0 ± 4.01 writhing episodes, confirming the robustness of the nociceptive stimulus. At both tested doses, all synthesized compounds significantly reduced the number of writhing episodes compared to the control group.

At 12.5 mg/kg, the mean number of writhes was 23.25 ± 5.14 for compound **5**, 17.0 ± 1.68 for compound **6**, 10.0 ± 1.53 for compound **7**, and 3.75 ± 0.48 for compound **8**, all of which were significantly lower than the control values (*p* < 0.001). In contrast, 12.5 mg/kg PCM (43.0 ± 2.55) did not differ significantly from the control group (*p* > 0.05), indicating a lack of peripheral antinociceptive activity at this dose.

At 25 mg/kg, all treatments significantly reduced the number of writhes compared to the control (*p* < 0.001). The observed mean values were 16.25 ± 4.05 for compound **5**, 16.25 ± 3.25 for compound **6**, 9.33 ± 2.40 for compound **7**, 8.75 ± 1.25 for compound **8**, and 6.75 ± 1.38 for PCM. At this higher dose, PCM exhibited a marked peripheral antinociceptive effect comparable to that of the synthesized compounds.

Direct comparisons with PCM at 12.5 mg/kg revealed that all compounds at the same dose were significantly more effective in reducing the number of writhes, with compound **8** demonstrating the greatest inhibitory effect. At 25 mg/kg, although all treatments—including PCM—produced robust inhibition of the writhing response, no statistically significant differences were detected between the compounds and PCM (*p* > 0.05), indicating a comparable level of peripheral antinociceptive efficacy at this dose.

### 3.8. Anti-Inflammatory Effect of Compounds ***5***–***8***

**Figure 6:**
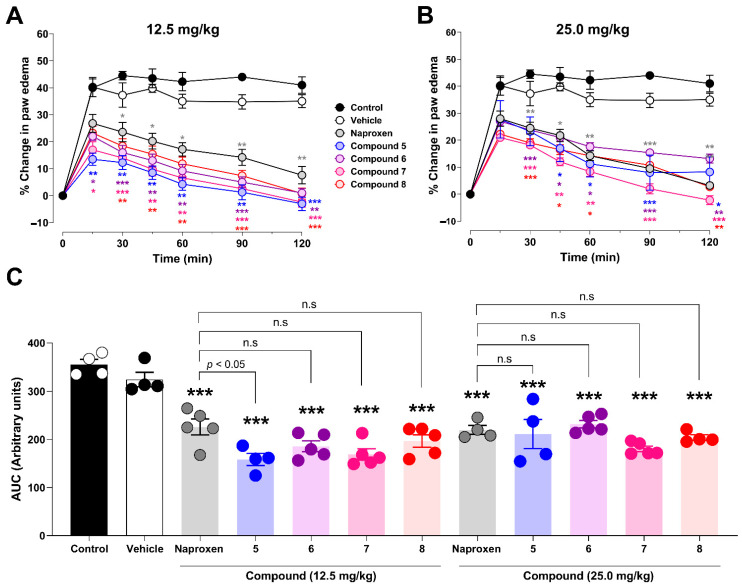
Anti-inflammatory activity of paracetamol-inspired analogs in a carrageenan-induced paw edema model. Acute inflammation was induced by carrageenan injection into the hind paw of *Rattus norvegicus* var. Holtzman, and paw edema was monitored over a 120 min experimental period. The animals received vehicle (Tween 80), naproxen (reference drug), or compounds **5**–**8**. Panel (**A**) shows the time course of paw edema following treatment with 12.5 mg/kg, whereas Panel (**B**) depicts the same treatment administered at 25 mg/kg. Panel (**C**) summarizes the anti-inflammatory response, expressed as the area under the curve (AUC) of paw edema over time. Edema is expressed as the change in paw volume relative to baseline. Data are presented as mean ± SEM (n = 4–5 animals per group). Statistical analysis was performed using two-way ANOVA, followed by Tukey’s multiple comparisons test. Statistical significance versus the control is indicated as * *p* < 0.05, ** *p* < 0.01, and *** *p* < 0.001.

The anti-inflammatory activity of the synthesized compounds was evaluated using the carrageenan-induced paw edema model at doses of 12.5 and 25 mg/kg (Figure 6). In the control group, carrageenan induced a robust inflammatory response, with paw edema increasing from 40.0 ± 3.2% at 15 min to 44.5 ± 1.6% at 30 min, remaining elevated at 90 min (44.0 ± 0.8%), and slightly decreasing at 120 min (41.0 ± 3.1%). A comparable temporal profile was observed in the vehicle-treated group.

Naproxen produced a significant and time-dependent reduction in paw edema compared with the control group from 30 min onward (*p* < 0.05–0.01), reaching 7.6 ± 3.3% at 120 min.

At 12.5 mg/kg, compounds **5**–**8** produced marked anti-inflammatory effects (Figure 6A). Early reductions in paw edema were observed at 15 min for compounds **5**–**7**, whereas compound **8** produced a smaller, non-significant reduction. From 30 min onward, all compounds significantly reduced paw edema compared to that in the control group (*p* < 0.01–0.001). By 90 min, edema values decreased to 1.25 ± 2.8% (compound **5**), 5.0 ± 1.9% (compound **6**), 2.6 ± 1.9% (compound **7**), and 7.4 ± 2.0% (compound **8**), compared to 44.0 ± 0.8% in the control group. At 120 min, compounds **5** and **7** produced near-complete suppression of edema (−3.0 ± 2.5% and −2.4 ± 1.8%, respectively), whereas compounds **6** and **8** showed minimal residual inflammation. No statistically significant differences were detected between naproxen and the tested compounds at this dose.

At 25 mg/kg, a similar time-dependent anti-inflammatory pattern was observed (Figure 6B). Naproxen significantly reduced paw edema from 30 min onward (*p* < 0.01–0.0001). Compounds **6**–**8** showed significant edema inhibition from 30 min, whereas compound **5** reached statistical significance at slightly later time points. At 90 min, paw edema values were reduced to 8.0 ± 6.5% (compound **5**) and 2.0 ± 1.9% (compound **7**) compared with 44.0 ± 0.8% in the control group. At 120 min, compound **7** produced near-complete suppression of edema (−2.2 ± 1.6%), while compounds **5** and **8** maintained low levels of residual inflammation. Overall, most compounds displayed anti-inflammatory effects comparable to those of naproxen, although compound **6** exhibited a significantly lower effect than naproxen at 120 min (*p* < 0.05).

To provide an integrated quantitative assessment, the area under the curve (AUC) of paw edema over the 120 min observation period was calculated (Figure 6C). At 12.5 mg/kg, all compounds significantly reduced AUC values compared with the control group (*p* < 0.0001), with compound **5** producing the greatest reduction (158.3 ± 12.7 a.u.), exceeding that of naproxen (225.8 ± 16.5 a.u.). At 25 mg/kg, compound **7** showed the most pronounced anti-inflammatory effect (180.5 ± 5.7 a.u.), followed by compounds **8** (204.1 ± 5.7 a.u.) and **5** (211.4 ± 30.2 a.u.), whereas naproxen showed a slightly higher AUC value (219.6 ± 9.2 a.u.). Collectively, these results identified compounds **5** and **7** as the most effective anti-inflammatory agents in this series.

### 3.9. Antipyretic Effect of Compounds ***5***–***8***

**Figure 7:**
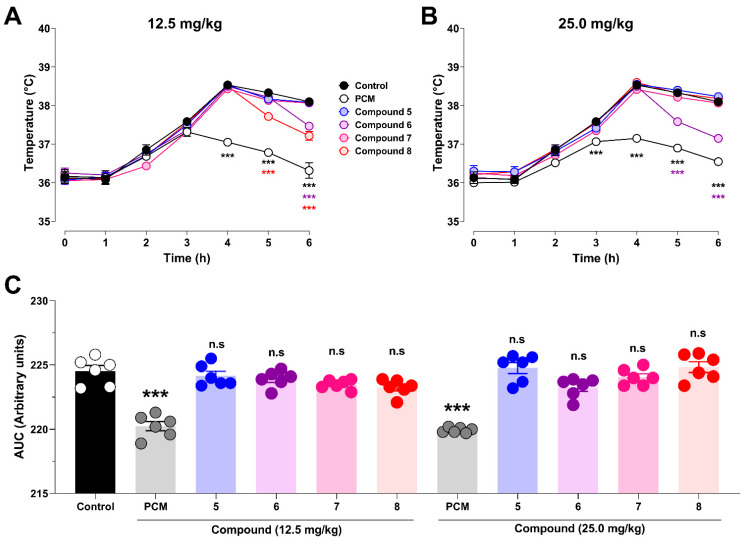
Antipyretic activity of paracetamol-inspired analogs in an LPS-induced fever model. Body temperature was monitored for 6 h following lipopolysaccharide (LPS) administration in *Rattus norvegicus* var. Holtzman. Panel (**A**) shows the temporal evolution of body temperature after treatment with PCM (paracetamol) and compounds **5**—at 12.5 mg/kg, while Panel (**B**) corresponds to the same treatments administered at 25 mg/kg. Panel (**C**) summarizes the antipyretic response quantified as the area under the temperature–time curve (AUC) over a 6 h observation period. Data are expressed as mean ± SEM (n = 6 animals per group). Statistical analysis was performed using two-way ANOVA, followed by Tukey’s multiple comparisons test. Statistical significance versus the control is indicated as *** *p* < 0.001.

Given that antipyretic activity is a hallmark pharmacological property of paracetamol and is mechanistically associated with the inhibition of prostaglandin synthesis, rectal temperature was monitored to assess whether the experimental analogs retained this effect in an LPS-induced fever model. As shown in Figure 7A, no significant differences in rectal temperature were observed among the treatment groups during the early phase of the experiment (0–3 h) following lipopolysaccharide (LPS) administration (*p* > 0.05). The control group developed a clear febrile response, reaching peak body temperatures at 4 and 5 h (38.53 ± 0.05 °C and 38.33 ± 0.06 °C, respectively), followed by a slight decline at 6 h (38.10 ± 0.04 °C).

At a dose of 12.5 mg/kg, PCM elicited a robust and sustained antipyretic effect from 4 h onward, significantly reducing rectal temperature compared to the control group (*p* < 0.001), with values of 37.05 ± 0.04 °C at 4 h, 36.78 ± 0.07 °C at 5 h, and 36.32 ± 0.20 °C at 6 h.

Among the experimental compounds, compound **8** significantly attenuated the febrile response at 5 h compared to the control group (37.72 ± 0.07 °C; *p* < 0.001). At 6 h, significant reductions in rectal temperature were observed for both compounds **6** and **8**, which lowered body temperature to 37.47 ± 0.06 °C and 37.22 ± 0.11 °C, respectively (*p* < 0.001 vs. control group). However, at later time points, all experimental compounds were significantly less effective than the PCM (*p* < 0.001).

Consistent with the findings at the lower dose, evaluation at 25 mg/kg (Figure 7B) revealed no significant differences among the treatments during the early phase of the assay (0–2 h). At 3 h post-LPS administration, PCM significantly reduced rectal temperature compared with the control group (37.07 ± 0.03 °C vs. 37.58 ± 0.09 °C, respectively; *p* < 0.001), confirming its expected antipyretic activity.

From 4 h onward, PCM (25 mg/kg) significantly attenuated the febrile response relative to the control group, coinciding with the peak temperatures observed in untreated animals. Among the experimental compounds, only compound **6** produced a significant antipyretic effect, which became evident at 5 h (37.58 ± 0.07 °C vs. 38.33 ± 0.06 °C in the control group; *p* < 0.001) and was maintained for 6 h (37.15 ± 0.10 °C vs. 38.10 ± 0.04 °C; *p* < 0.001). However, this effect was less pronounced than that of PCM, which reduced the rectal temperature to 36.90 ± 0.07 °C at 5 h and 36.55 ± 0.06 °C at 6 h.

To integrate the antipyretic response over time, the area under the temperature–time curve (AUC) was calculated for each treatment during the 6 h period following LPS-induced pyrexia (Figure 7C). At 12.5 mg/kg, PCM produced a marked and statistically significant reduction in AUC (220.3 ± 0.36) compared to the control group (224.5 ± 0.43; *p* < 0.0001). In contrast, compounds **5** (224.2 ± 0.35), **6** (223.9 ± 0.26), **7** (223.5 ± 0.15), and **8** (223.3 ± 0.26) did not differ significantly from the control group and showed no significant differences compared to PCM at this dose.

Similarly, at 25 mg/kg, PCM significantly reduced the AUC (219.9 ± 0.08; *p* < 0.0001 vs. control), whereas none of the experimental compounds—compounds **5** (224.8 ± 0.43), **6** (223.3 ± 0.32), **7** (224.1 ± 0.26), or **8** (224.8 ± 0.42)—exhibited a significant reduction in AUC compared with the control group or achieved an effect comparable to PCM at the same dose.

Overall, these results indicate that despite transient reductions in body temperature at specific time points for certain compounds, only PCM produced a sustained and statistically significant antipyretic effect when evaluated using AUC in the LPS-induced fever model.

To provide an objective basis for compound prioritization, pharmacological endpoints were normalized on a 0–1 scale using min–max normalization across compounds **5**–**8**, integrating peripheral antinociception (writhing inhibition), anti-inflammatory activity (edema AUC reduction), central antinociception (hot plate response), and antipyretic response (temperature AUC reduction). Using dose-averaged values, compound **7** showed the highest integrated balance across endpoints, followed by compound **8**, which was driven by strong peripheral and central activity but a lower anti-inflammatory contribution. Compounds **5** and **6** exhibited more endpoint-restricted profiles. Detailed normalized values are provided in Tables S3 and S4 (Supplementary Material).

## 4. Discussion

This study describes the solvent-free synthesis and phenotypic pharmacological characterization of a series of 4-hydroxyphenylamino-naphthoquinones designed as paracetamol-inspired analogs. In this context, the term “paracetamol-inspired” refers specifically to the incorporation of the 4-hydroxyaniline pharmacophore and the pursuit of non-opioid analgesic activity, rather than replication of the metabolic pathways or molecular mechanisms of PCM. By integrating sustainable synthetic chemistry, in silico tools, and in vivo phenotypic evaluation, the present study identified a naphthoquinone-based scaffold displaying a consistent peripheral anti-inflammatory analgesic profile. While naphthoquinone derivatives, including amino-substituted analogs, have been widely explored, their evaluation has mainly focused on cytotoxic or redox-related activities. In contrast, the present study examines this scaffold within a phenotypic framework of inflammation-driven analgesia, differentiating it from previously reported naphthoquinone-based compounds.

The solvent-free, silica-assisted Michael addition to naphthoquinones employed in this study represents an efficient and environmentally benign synthetic strategy, providing significantly reduced reaction times and improved isolated yields compared to conventional conditions [25,26]. The acceleration observed is consistent with the dual role of silica gel as a heterogeneous catalytic surface and adsorption medium, facilitating reactant proximity and stabilizing polar transition states [47,48]. The regioselectivity observed for most derivatives highlights the robustness of this methodology. In contrast, the formation of an inseparable regioisomer mixture in compound **8** reflects the electronic and steric constraints imposed by the substituted naphthoquinone core. Conventional chromatographic attempts to isolate individual regioisomers consistently resulted in co-elution, indicating similar polarity and chromatographic behavior. Consequently, compound **8** was experimentally evaluated as a structurally characterized regioisomeric mixture (~2:1). Because the compound was tested as a mixture, computational analyses performed on the individual regioisomers are interpreted only as qualitative descriptors and not as direct representations of the biological behavior of the mixture.

The in silico analyses included in this study are interpreted strictly as hypothesis-generating tools that provide structural and physicochemical context to the observed pharmacological phenotype, rather than as evidence of direct target engagement. PASS-based predictions suggested antinociceptive and anti-inflammatory activities consistent with a non-opioid analgesic profile. However, because these models rely on global molecular descriptors, associations with specific pathways should be considered exploratory rather than mechanistically definitive.

DFT calculations revealed the electronic properties of the synthesized compounds, showing lower chemical hardness and higher electron affinity [49,50] than paracetamol, consistent with the redox-active nature of the naphthoquinone core. These features suggest enhanced molecular polarizability and a greater propensity for intermolecular interactions, including hydrogen bonding and π-driven contacts; however they do not define specific biological targets. 

Similarly, molecular docking suggested favorable ligand–target complementarity; however, these results represent structural hypotheses regarding binding feasibility and do not demonstrate functional activity or target modulation. In particular, the predicted interactions with CB1 and TRPV1 are considered speculative and are not used to support mechanistic claims in this study. In contrast, the overall consistency between docking outcomes and the anti-inflammatory profile supports further consideration of COX-related pathways, although experimental validation is required. Furthermore, no experimental evidence currently supports the formation of N-arachidonoyl conjugates for these compounds; therefore, any analogy with paracetamol-derived metabolites should be interpreted with caution. 

From a pharmacological perspective, the in vivo data provide a coherent and internally consistent profile that supports a predominantly peripheral, anti-inflammatory mechanism of action. All the compounds produced strong inhibition of acetic acid-induced writhing, a model associated with peripheral nociception mediated by inflammatory mediators such as prostaglandins, bradykinin, and cytokines [51,52,53]. Notably, at 12.5 mg/kg, the synthesized compounds were markedly more effective than paracetamol, indicating enhanced peripheral antinociceptive activity within this series. The selected doses (12.5–25 mg/kg) were based on commonly reported ranges for reference analgesics, such as paracetamol and naproxen, allowing for a comparative evaluation under similar experimental conditions. At this stage, the study was designed to identify phenotypic activity rather than to establish optimized dosing or pharmacokinetic parameters.

This interpretation is further supported by the carrageenan-induced paw edema model, in which all compounds produced rapid and sustained anti-inflammatory effects comparable to those of naproxen [54,55,56]. Because the late phase of carrageenan-induced inflammation is driven predominantly by prostaglandin production, the observed inhibition strongly supports the involvement of cyclooxygenase-related pathways. Taken together, the convergence of these two independent in vivo models suggests a predominantly peripheral mechanism consistent with anti-inflammatory and antinociceptive profiles linked to cyclooxygenase inhibition, which is proposed here as the primary working hypothesis for this series.

In contrast, central antinociceptive activity evaluated in the hot plate test was moderate and variable. Although compounds **5** and **8** produced measurable increases in nociceptive latency, these effects were less consistent than the peripheral responses and did not show a clear dose-dependent relationship. This observation is consistent with the predicted limited blood–brain barrier permeability and suggests that central effects, when present, may arise from partial CNS exposure or indirect modulation rather than primary central mechanisms [57]. These observations should be interpreted in light of the exploratory nature of this study. The use of n = 5 per group, while standard for preliminary phenotypic screening, may limit the detection of subtle differences in variable models, such as the hot plate and LPS-induced fever assays.

Antipyretic evaluation revealed a clear pharmacological dissociation from PCM. While PCM produced sustained reductions in body temperature, the synthesized compounds exhibited only transient effects and did not significantly reduce AUC. Because LPS-induced fever depends on central prostaglandin E_2_ synthesis within the hypothalamus, sustained antipyretic activity requires effective modulation of central pathways [58,59,60]. Therefore, the absence of sustained antipyretic effects further supports a predominantly peripheral mode of action and suggests limited engagement of central thermoregulatory mechanisms.

Normalization-based multicriteria analysis integrating peripheral antinociceptive, anti-inflammatory, central, and antipyretic endpoints identified compound **7** as the most balanced candidate within the series. This prioritization reflects its consistent performance in inflammation-driven models and supports its selection for further development.

In general, this study should be interpreted as a phenotype-driven identification of a bioactive scaffold rather than a target-specific mechanistic investigation. In addition, the safety assessment in the present study was limited to in silico ADMET predictions and therefore requires further experimental validation. The 4-hydroxyphenylamino-naphthoquinone framework emerges as a promising platform for the development of non-opioid, peripheral anti-inflammatory analgesics. Future studies should focus on experimental validation of the proposed COX-linked mechanism, pharmacokinetic characterization, and structure–activity relationship optimization to further define the therapeutic potential of this scaffold.

## 5. Conclusions

In summary, 4-hydroxyphenylamino-naphthoquinones, synthesized using a solvent-free methodology, were identified as phenotypically active scaffolds with robust antinociceptive and anti-inflammatory effects in inflammation-driven models. The in vivo data suggest a predominantly peripheral mechanism, consistent with anti-inflammatory and antinociceptive profiles linked to cyclooxygenase inhibition.

Compound **7** emerged as the most balanced candidate, whereas compound **8**, evaluated as a regioisomeric mixture, showed a stronger central antinociceptive component. In contrast to paracetamol, this series exhibited limited and non-sustained antipyretic activity.

Overall, these findings position the 4-hydroxyphenylamino-naphthoquinone framework as a promising non-opioid scaffold for peripheral inflammatory pain. Further studies are warranted to validate the underlying molecular mechanisms.

## Figures and Tables

**Table 1:** Study Design for Pharmacological Analysis.

Group	Treatment Allocation			p.o. Dose (mg/kg)
	Antinociceptive	Anti-Inflammatory	Antipyretic	
control	vehicle	vehicle	vehicle	-
reference standard	PCM	NPX	PCM	12.5 and 25.0
test groups	compounds **5**–**8**	compounds **5**–**8**	compounds **5**–**8**	12.5 and 25.0

PCM: paracetamol; NPX: naproxen.

**Table 2:**
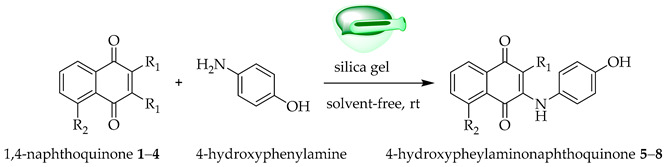
Yields and reaction times of 4-hydroxyphenylaminonaphthoquinones **5**–**8** synthesized under solvent-free conditions.

Entry Nº	R_1_	R_2_	Solvent-Free		Product N°
			Yield (%)	Time	
**1**	H	H	89.0	0.5 h	**5**
**2**	Cl	H	78.7	1.0 h	**6**
**3**	H	OH	50.0	2.0 h	**7**
**4**	H	NO_2_	89.7	8.0 h	**8** *

* Compound **8** was obtained as a regioisomeric mixture.

**Table 3:** PASS prediction results for compounds **5**–**8** and PCM.

Biological Activity	Compound									
	5		6		7		8		PCM	
	Pa	Pi	Pa	Pi	Pa	Pi	Pa	Pi	Pa	Pi
**Antinociceptive**	0.345	0.149	0.269	0.208	–	–	–	–	0.413	0.099
Analgesic stimulant	0.214	0.169	0.220	0.156	–	–	–	–	0.278	0.060
Vanilloid 1 agonist	0.212	0.074	0.396	0.029	0.335	0.063	0.267	0.142	0.466	0.011
**Anti-inflammatory**	0.427	0.082	0.347	0.124	0.411	0.090	0.239	0.226	0.319	0.144
Cyclooxygenase 1 inhibitor	0.151	0.036	0.087	0.068	0.138	0.041	–	–	0.094	0.062
Cyclooxygenase 3 inhibitor	0.102	0.016	0.112	0.013	0.082	0.023	0.063	0.040	0.036	0.004
**Antipyretic**	0.435	0.021	0.279	0.054	0.421	0.023	0.262	0.064	0.675	0.005
Cyclooxygenase inhibitor	0.133	0.060	–	–	0.110	0.076	–	–	0.294	0.019

PASS predictions for compound **8** correspond to both regioisomers, which produced identical Pa/Pi values in the PASS algorithm. PCM: paracetamol; Pa: probability of activity; Pi: probability of inactivity.

**Table 4:** Quantum chemical descriptors of compounds **5**–**7**, the two regioisomers of compound **8**, and the reference drug paracetamol (PCM) calculated at the B3LYP/6-311G(d,p) level of theory. I = ionization potential, A = electronic affinity, μ = (E_HOMO_ + E_LUMO_)/2 (chemical potential), and η = (E_LUMO_ − E_HOMO_)/2 (chemical hardness).

Compound	Isomer	I (kcal·mol^−1^)	A (kcal·mol^−1^)	µ (kcal·mol^−1^)	η (kcal·mol^−1^)
**5**	−	134.6	72.4	−103.5	31.1
**6**	−	137.3	75.8	−106.5	30.8
**7**	−	133.9	69.7	−101.8	32.1
**8** *	8-nitro (minor)	136.8	79.0	−107.9	28.9
	5-nitro (major)	137.0	78.2	−107.6	29.4
**PCM**	−	134.8	12.2	−73.5	61.3

* Compound **8** was obtained as an inseparable regioisomeric mixture (~66:34, major:minor). DFT descriptors were calculated independently for each regioisomer and are reported separately. PCM: paracetamol.

**Table 5:** Free binding energy results (in kcal.mol^−1^) from molecular docking calculations of compounds **5**–**7**, the two regioisomers of compound **8**, PCM and AM404 with COX-2 enzyme (5IKR).

Compound	Isomer	ΔE_Bind_ (kcal.mol^−1^)
		COX-2 Enzyme
		5IKR
**5**	–	−9.9
**6**	–	−9.7
**7**	–	−9.7
**8** *	8-nitro (minor)	−9.9
	5-nitro (major)	−9.7
**PCM**	–	−6.1
**AM404**	–	−8.2

* Compound **8** was obtained as an inseparable regioisomeric mixture (~66:34, major:minor). Free binding energies were calculated independently for each regioisomer and are reported separately. Positive controls: PCM (paracetamol) and AM404 (*N*-arachidonoylphenolamine).

**Table 6:** ADMET properties of compounds **5**−**7**, the two regioisomers of compound **8**, and PCM.

N°	Property									
	Absorption			Distribution			Metabolism	Excretion	Toxicity	
	Caco–2	IA	SP	VDss	BBB	CNS	CYP2D6/CYP3A4 Inhibitor	TC	Oral Rat Acute Tox. (LD_50_)	Oral Rat Chronic Tox.—LOAEL
**5**	1.024	93.804	−3.272	−0.334	−0.022	−2.037	No/No	0.137	2.305	2.086
**6**	1.018	92.341	−2.817	−0.390	−0.023	−1.929	No/Yes	−0.060	2.241	1.940
**7**	1.038	90.919	−3.611	−0.513	−0.684	−2.227	No/No	0.049	2.300	1.991
**8** *	0.546	89.118	−2.749	−0.050	−0.216	−2.217	No/No	0.042	2.770	1.831
**8** **	0.516	89.045	−2.763	−0.084	−0.216	−2.215	No/Yes	0.040	2.813	1.905
**PCM**	1.231	93.858	−2.832	−0.242	−0.209	−2.319	No/No	0.487	2.166	2.636

Compound **8** was obtained as an inseparable regioisomeric mixture (~66:34, major:minor). ADMET properties were calculated independently for each regioisomer and are reported separately: **8** *: minor regioisomer; **8** **: major regioisomer. **Caco–2**: Caucasian colon adenocarcinoma permeability (Log Papp in 10^−6^ cm/s). **IA**: intestinal absorption (% absorbed). **SP**: skin permeability (log Kp). **VDss**: steady-state volume of distribution (Log L/kg). **BBB**: blood–brain barrier permeability (log BB). CNS: central nervous system (log PS). **CYP2D6**: cytochrome P_450_ 2D6 inhibitor; **CYP3A4**: cytochrome P_450_ 3A4 inhibitor. **TC**: total clearance (Log mL/min/kg). **LD_50_**: lethal dose, 50% (mol/kg). **LOAEL**: lowest observed adverse effect level (log mg/kg bw/day).

## Data Availability

Data are contained within the article and Supplementary Materials.

## Supplementary Materials

The following supporting information can be downloaded at: https://www.mdpi.com/article/10.3390/pharmaceutics18040482/s1, Table S1: Solvents and reagents used in this study, including CAS numbers, molecular weight and purity; Figures S1 and S2: ^1^H-NMR and ^13^C-NMR for compound **5**; Figures S3 and S4: ^1^H-NMR and ^13^C-NMR for compound **6**; Figures S5–S7: ^1^H-NMR, ^13^C-NMR and HMBC for compound **7**; Figure S8–S11: ^1^H-NMR, ^13^C-NMR, HMQC and HMBC for compound **8**; Figures S12 and S13: ^1^H-NMR and ^13^C-NMR for compound 5-nitro-1,4-naphthoquinone; Table S2: Free binding energy results (in kcal.mol^−1^) from molecular docking calculations of compounds **5**–**7**, the two regioisomers of compound **8**, PCM and AM404 with proteins TRPV1 receptor (3J5P) and CB1 receptor (5U09); Figures S14 and S15: Representative docking poses and interaction profiles of compounds **5**–**7**, the two regioisomers of compound **8**, and paracetamol (PCM) within the TRPV1 and CB1 receptors binding site; Figure S16: The optimized structures of compounds **5**–**7**, the two regioisomers of compound **8**, and the reference drug paracetamol (PCM). Table S3: Dose-averaged raw pharmacological values used for normalization; Table S4: Normalized Pharmacological parameters of compounds **5**–**8**.
