# Supplementary material for: Synthesis of 4-Hydroxyphenylamino-Naphthoquinones as Paracetamol-Inspired Analogs: Chemical, In Silico, and Phenotypic Pharmacological Evaluation
Source: Pharmaceutics. 2026 Apr 14;18(4):482. doi: 10.3390/pharmaceutics18040482 (PMC13119072; doi:10.3390/pharmaceutics18040482)
Supplement: Supplementary file 1 [file pharmaceutics-18-00482-s001.zip › pharmaceutics-4217868-supplementary.pdf]

Synthesis of 4-Hydroxyphenylamino-Naphthoquinones as Paracetamol-Inspired Analogs: Chemical, *In Silico*, and Phenotypic Pharmacological Evaluation

Iván M. Quispe-Díaz [1,*], Oswaldo Rebaza-Rioja [1], Sussan Lopez-Mercado [2], Cinthya Enriquez-Lara [3], Daniel Asunción-Alvarez [1], Roberto O. Ybañez-Julca [1], Elena Mantilla-Rodríguez [1], Wilfredo O. Gutiérrez-Alvarado [4], Ricardo Pino-Rios [5], Jaime A. Valderrama [2], and Julio Benites [2,3*]

[1] Facultad de Farmacia y Bioquímica, Universidad Nacional de Trujillo, Trujillo 13011, Perú; iquispe@unitru.edu.pe (I.M.Q.-D); orebaza@unitru.edu.pe (O.R.-R.); hasuncion@unitru.edu.pe (D.A.-A.); rybanez@unitru.edu.pe(R.O.Y-J.); amantilla@unitru.edu.pe (E.M.-R)

[2] Química y Farmacia, Facultad de Ciencias de la Salud, Universidad Arturo Prat, Casilla 121, Iquique 1100000, Chile; sussan.j.lopez.m@gmail.com (S.L.-M.); jaimeadolfov@gmail.com (J.A.V); juliob@unap.cl (J.B.).

[3] Programa de Doctorado en Química Medicinal, Facultad de Ciencias de la Salud, Universidad Arturo Prat, Casilla 121, Iquique 1100000, Chile; cenriquez@estudiantesunap.cl (C.E.)

[4] Facultad de Farmacia y Bioquímica, Universidad Nacional de la Amazonía Peruana, Iquitos 16001, Perú; wilfredo.gutierrez@unapiquitos.edu.pe (W.O.G.-A)

[5] Departamento de Ciencias Químicas, Facultad de Ciencias Exactas, Universidad Andrés Bello, Santiago 8370146, Chile; ricardo.pino.r@unab.cl (R.P.-R)

* Correspondence: iquispe@unitru.edu.pe (I.M.Q.-J.); juliob@unap.cl (J.B.); Tel.: +51-937-696-623 (I.M.Q.-D.); Tel.: +56-9-98277790 (J.B.)

Table S1. Solvents and reagents used in this study, including CAS numbers, molecular weight and purity.

**solvents and reagents**	**CAS number**	**Molecular weight**	**Purity (%)**
Silica gel	112926-00-8	60.08	99,0
DMSO	67-68-5	78.13	≥99.7
$CDCl_3$	865-49-6	120,38	≥99,0
4-hydroxyaniline	123-30-8	109.13	>98,0
1,4-naphthoquinones	130-15-4	158.15	>97,0
Paracetamol	103-90-2	151.16	>99,0
Tween 80	9005-65-6	76 kDa[3]	70% oleic acid
Acetic acid	64-19-7	60.05	99,99
Carrageenan	9000-07-1	400-560 kDa	99,0
Naproxen	22204-53-1	230.26	<=100,0

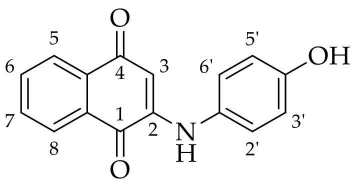

Compound 5

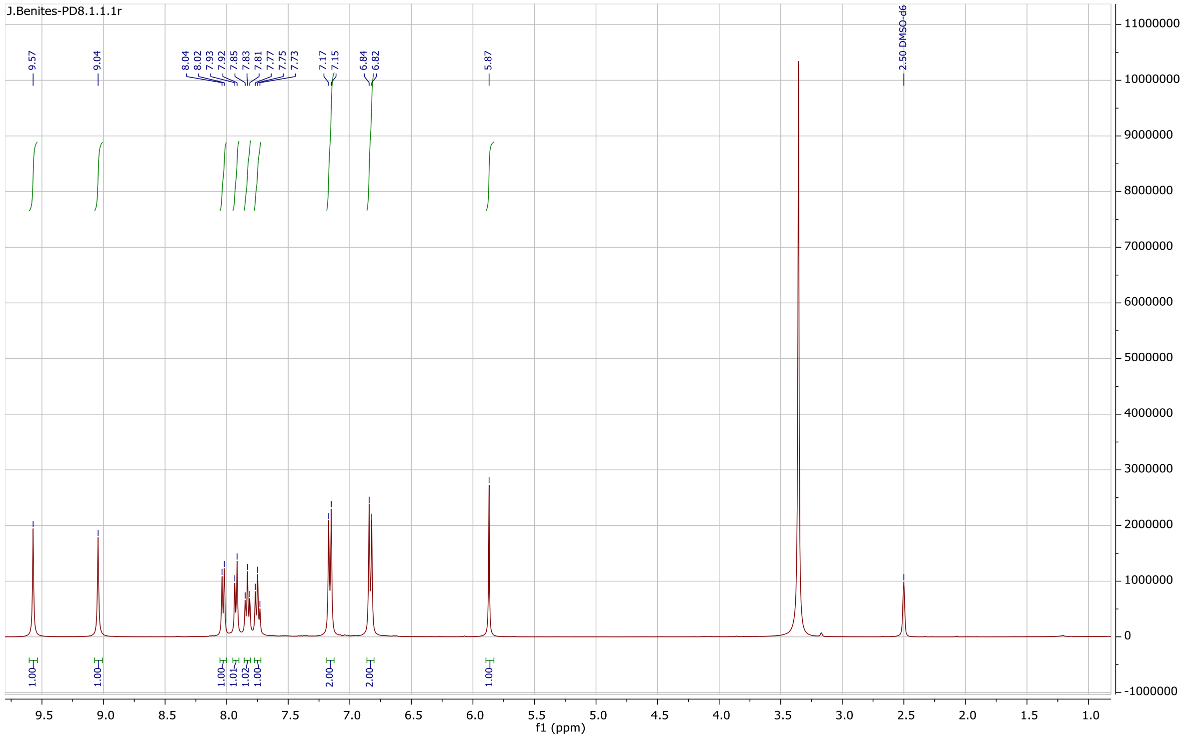

Figure S1. ^{1}H-NMR for compound 5.

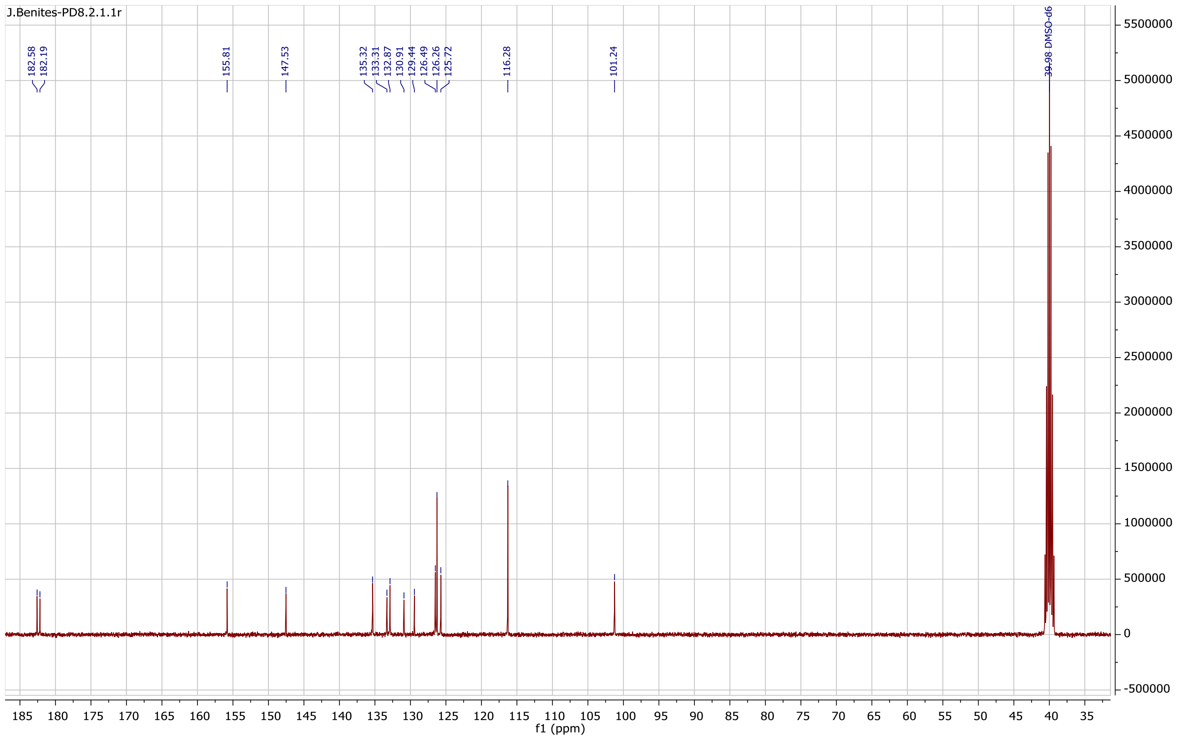

Figure S2. ^{13}C-NMR for compound 5.

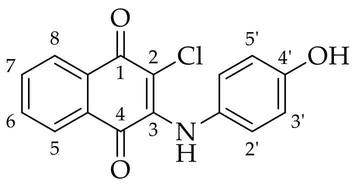

Compound 6

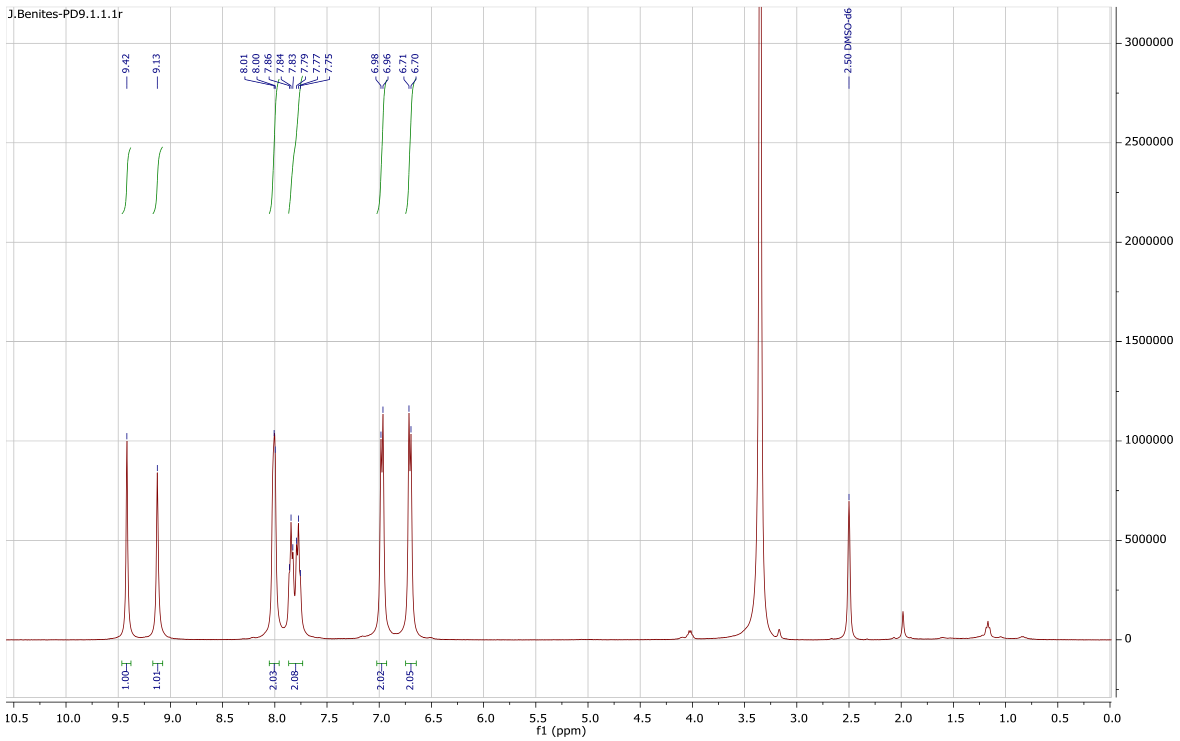

Figure S3. ^{1}H-NMR for compound 6.

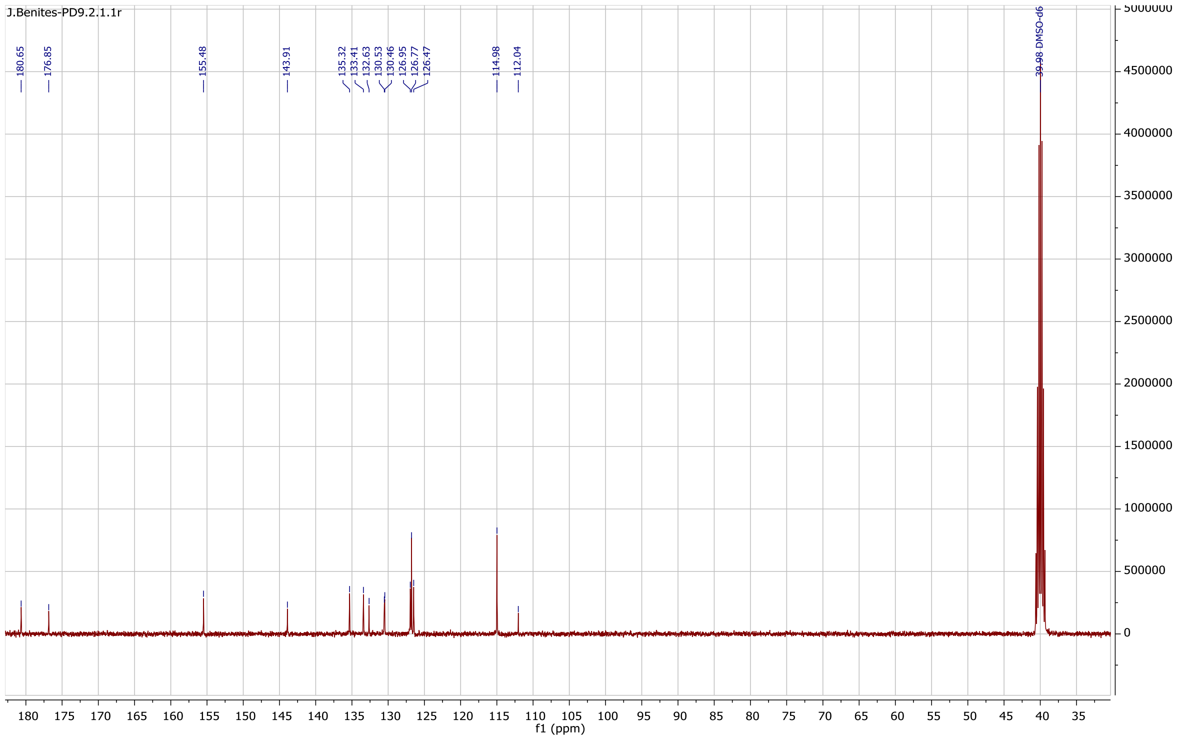

Figure S4. ^{13}C-NMR for compound 6.

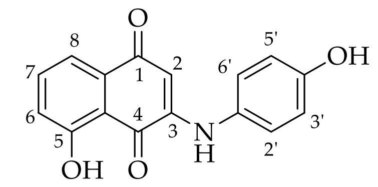

Compound 7

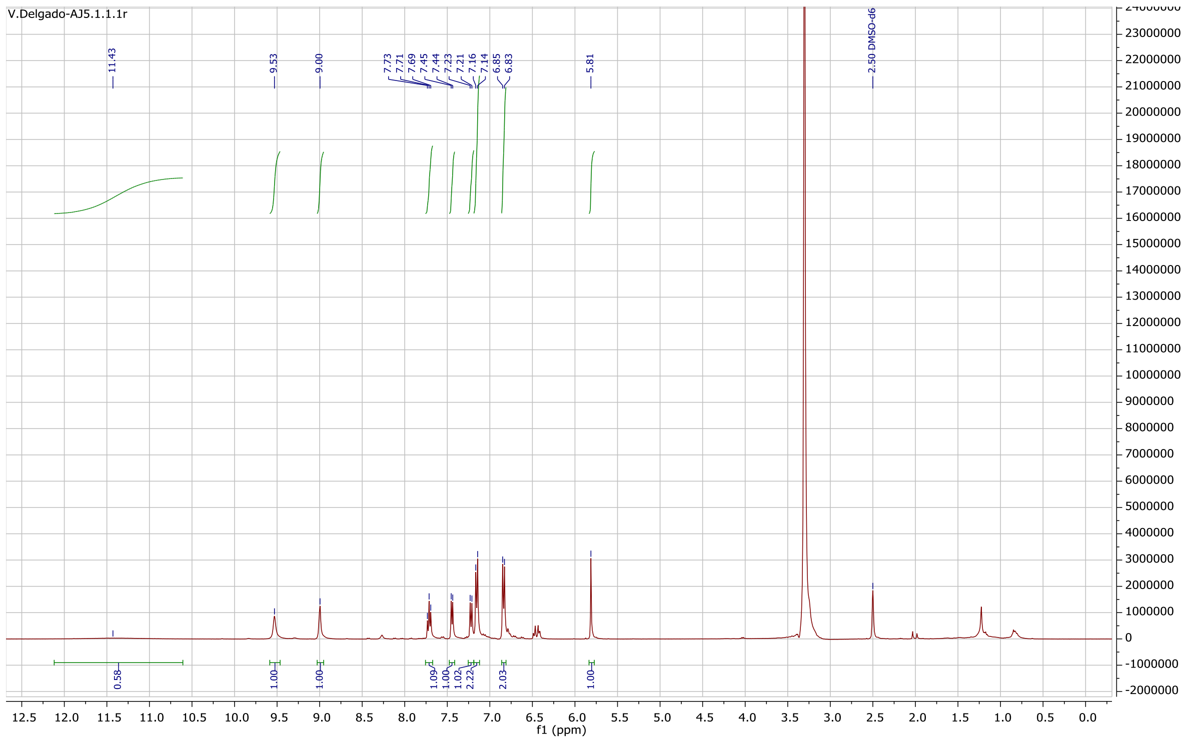

Figure S5. ¹H-NMR for compound 7.

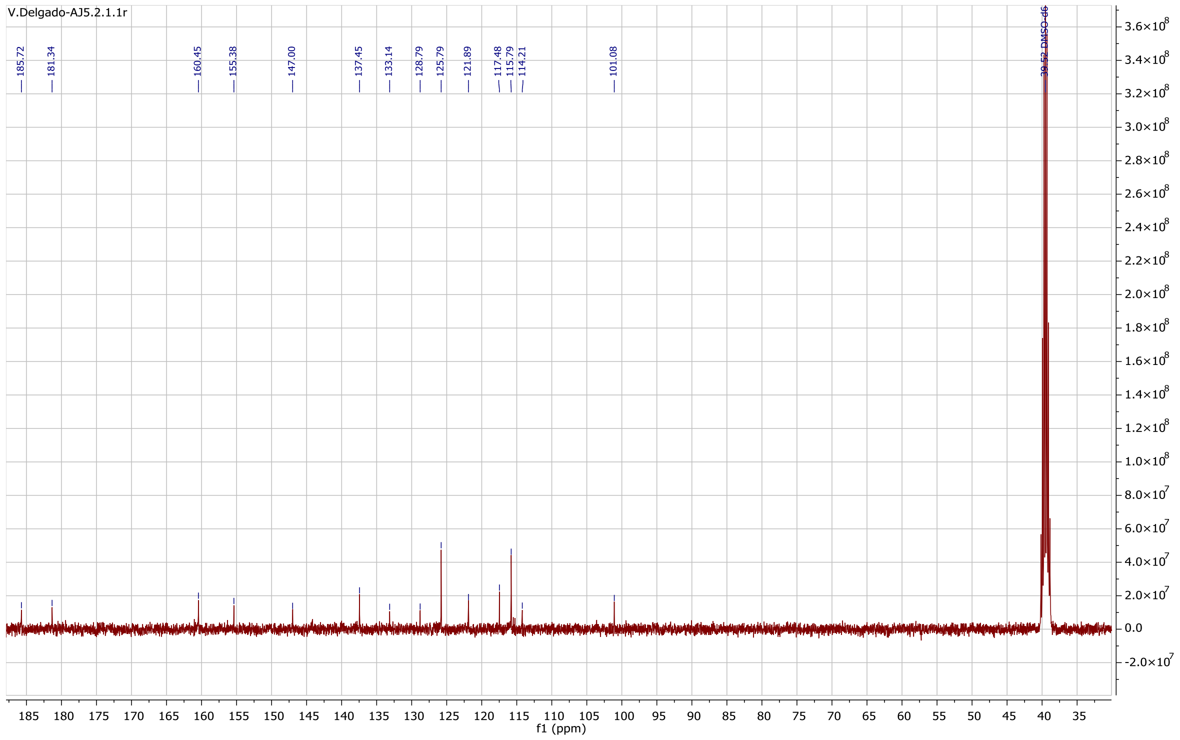

Figure S6. ^{13}C-NMR for compound 7.

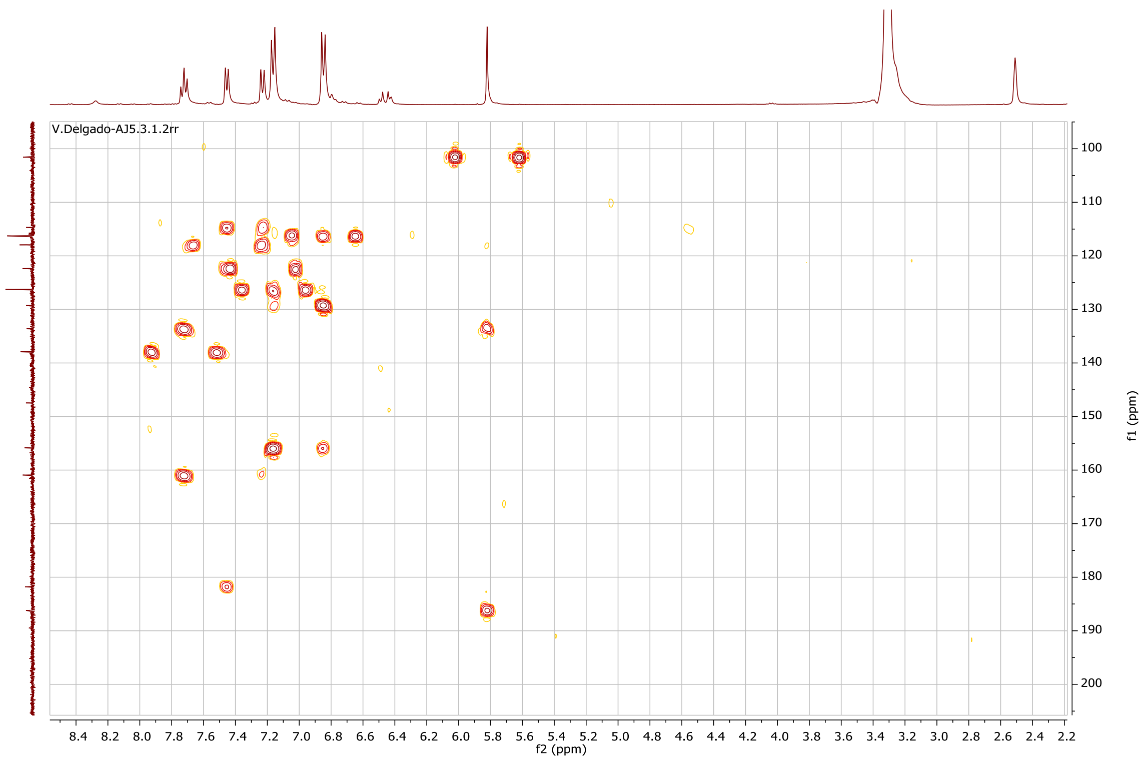

Figure S7. HMBC for compound 7.

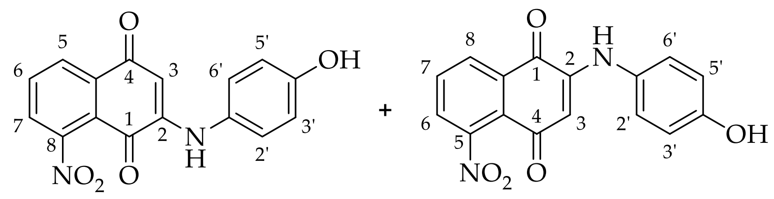

Compound 8

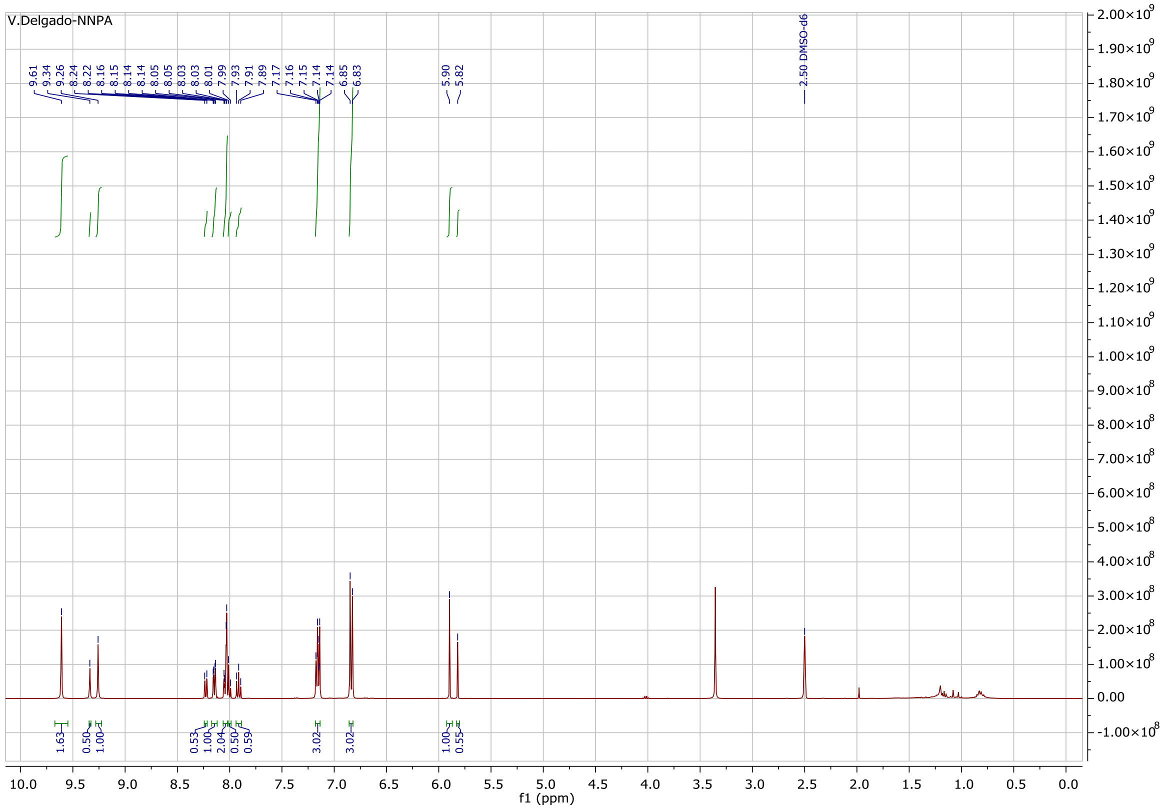

Figure S8. ¹H-NMR for compound 8.

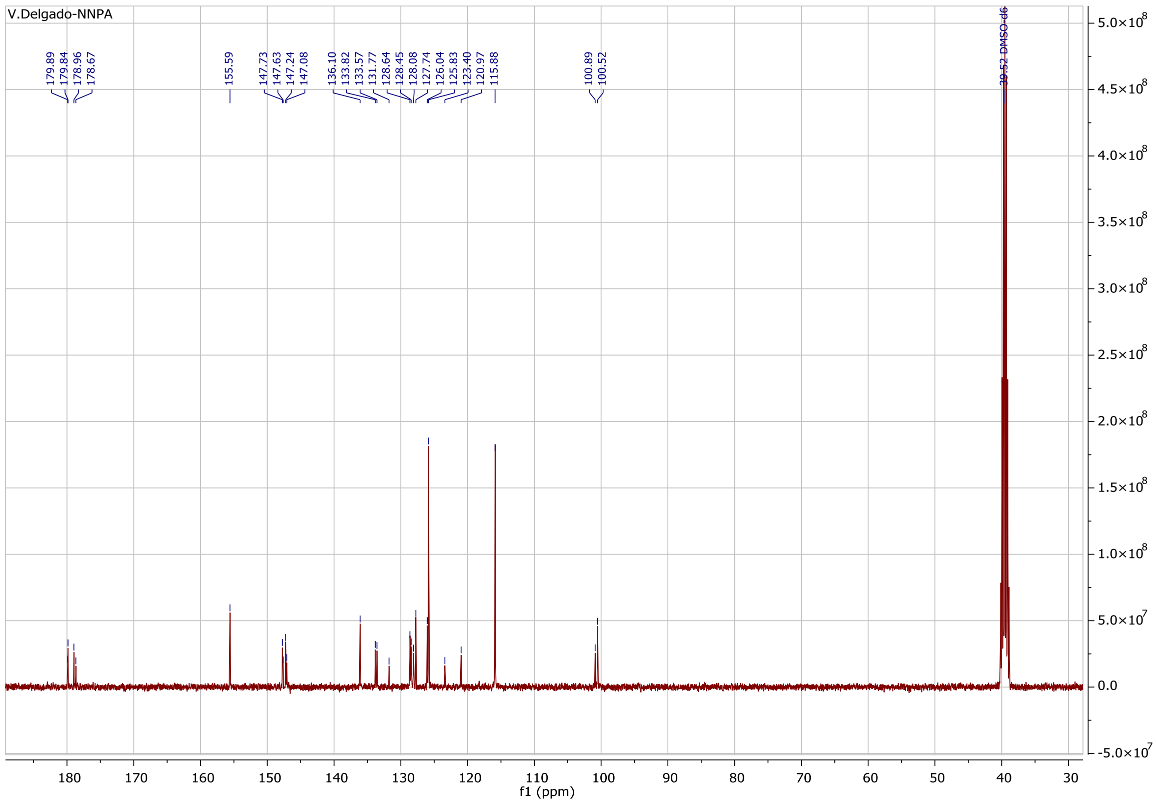

Figure S9. ^{13}C-NMR for compound 8.

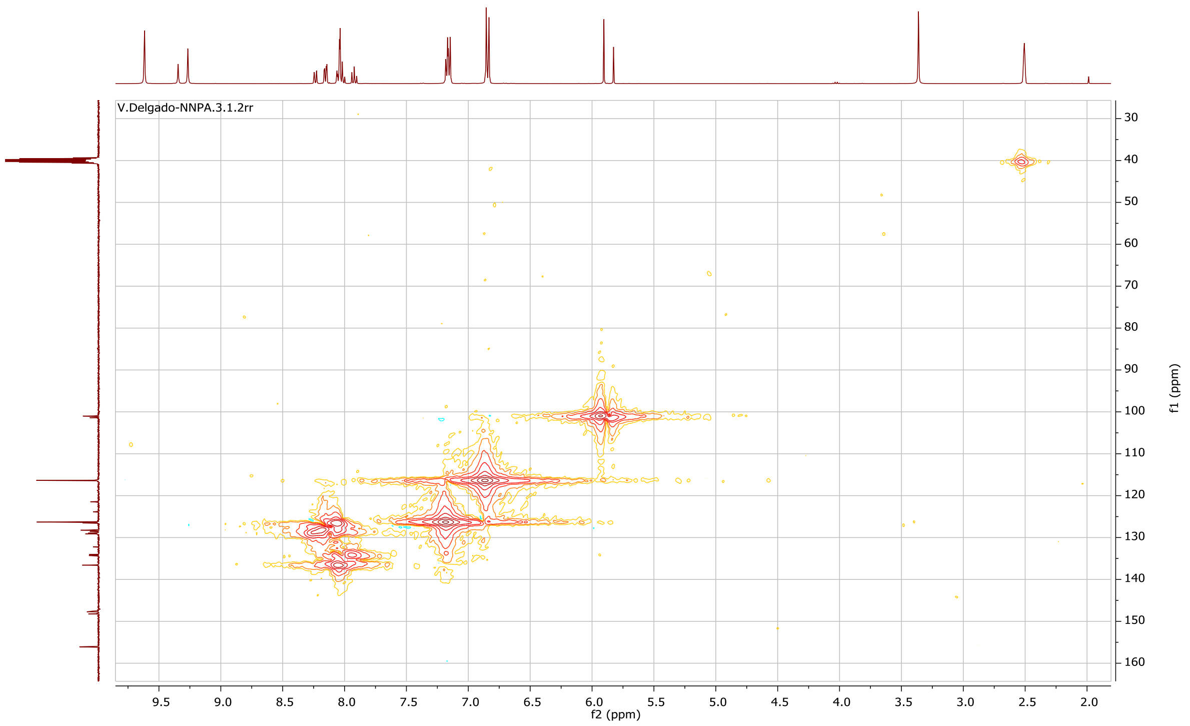

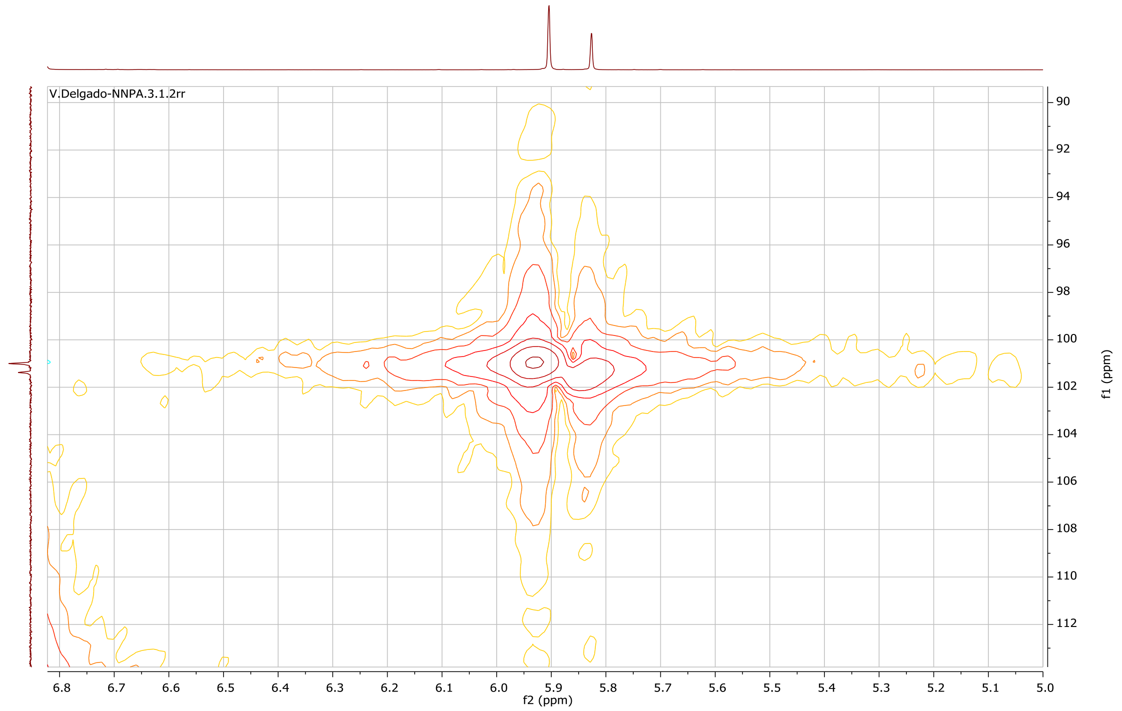

Figures S10. HMQC for compound 8.

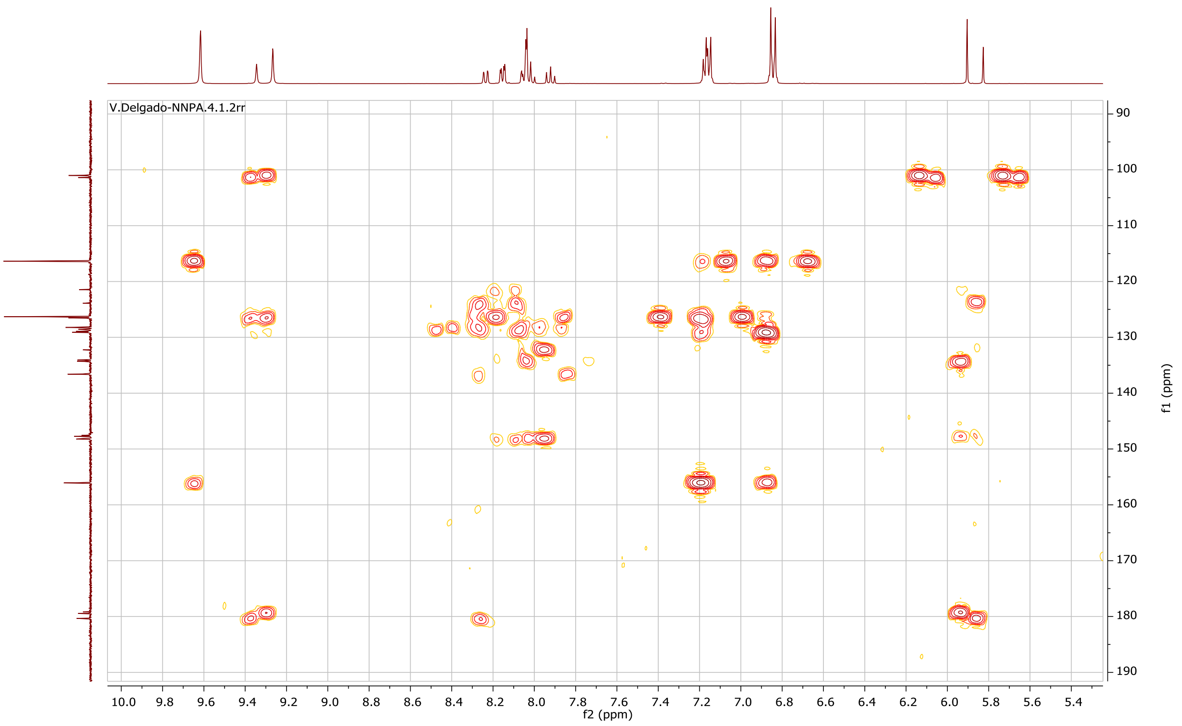

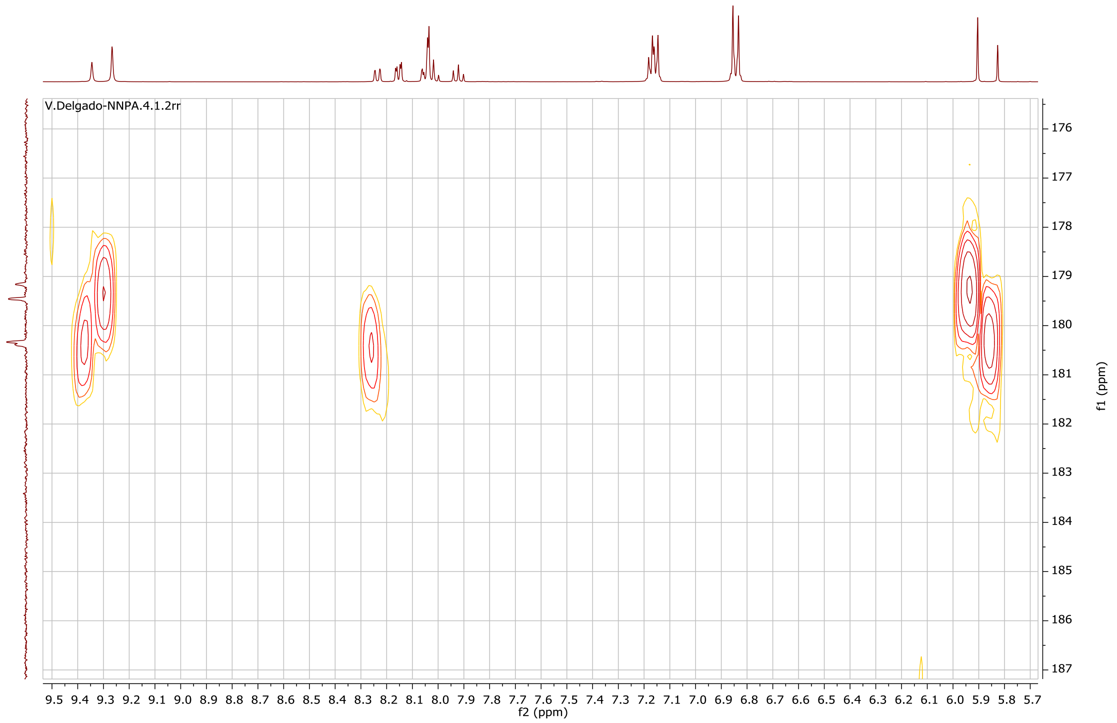

Figures S11. HMBC for compound 8.

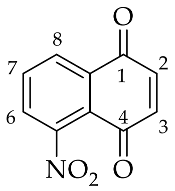

5-nitro-1,4-naphthoquinone

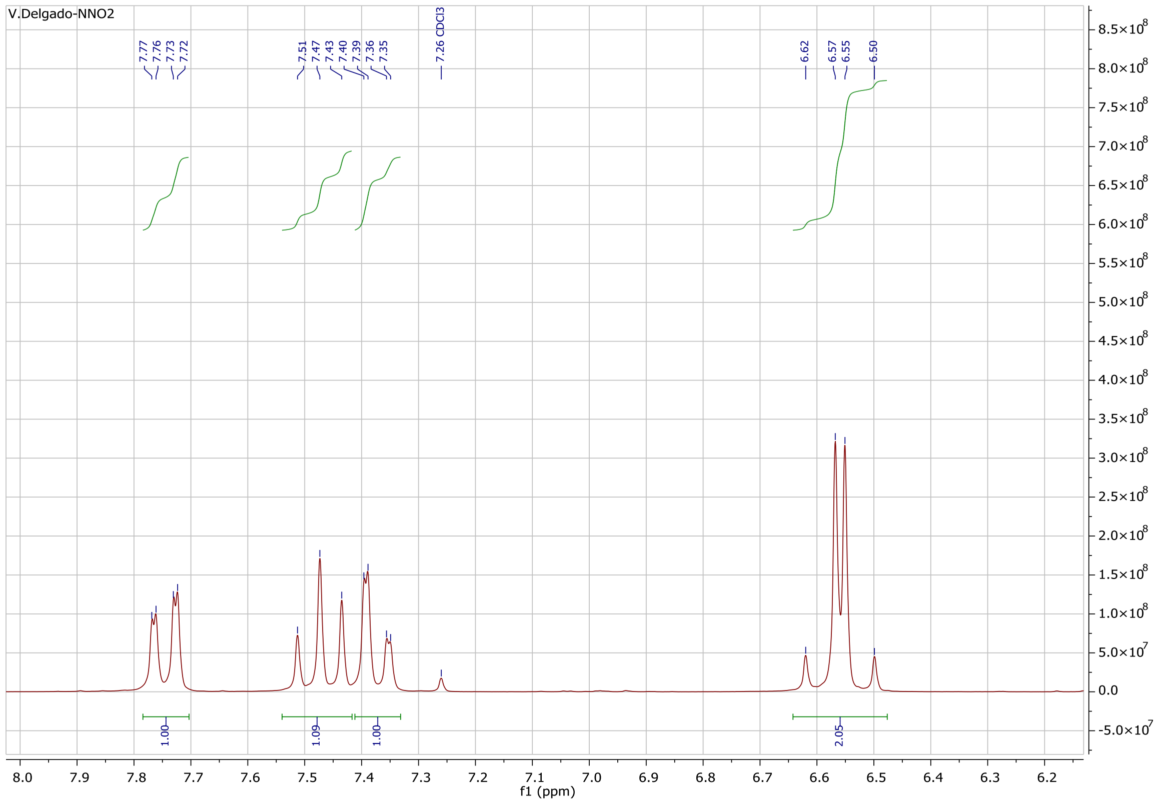

Figure S12. ¹H-NMR for compound 5-nitro-1,4-naphthoquinone.

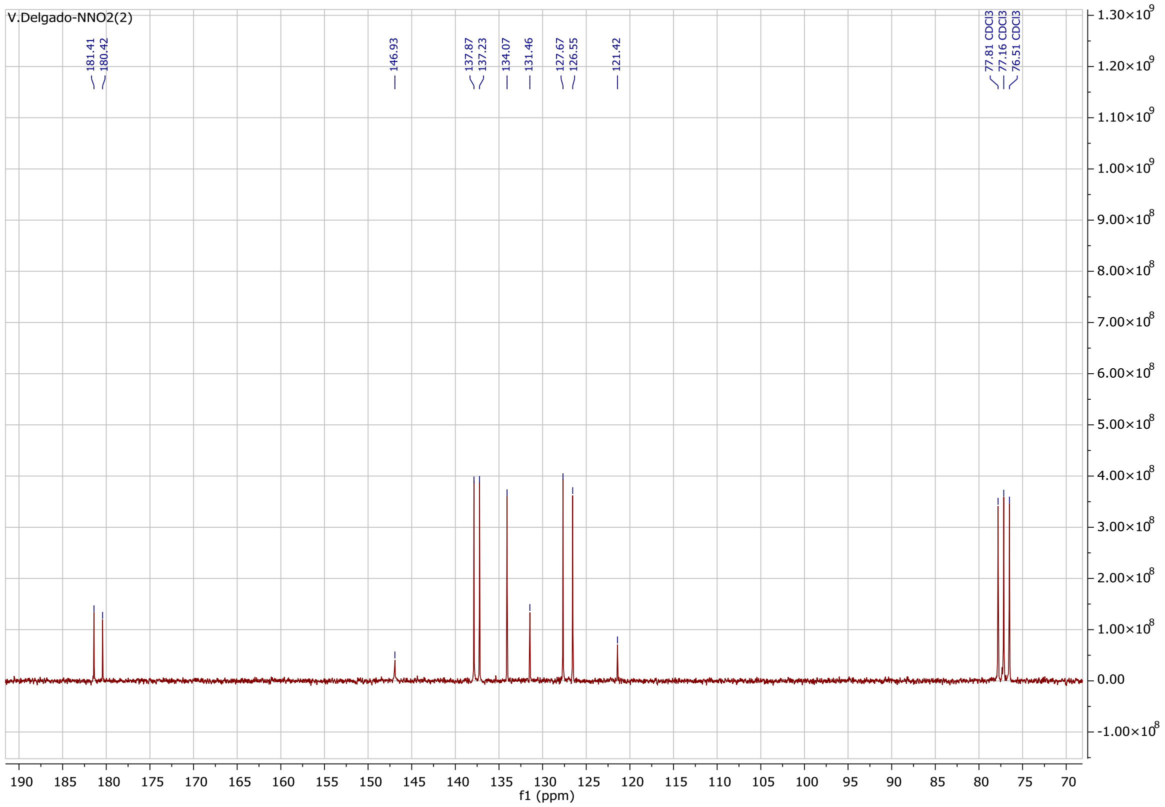

Figure S13. **^{13}C-NMR for compound: 5-nitro-1,4-naphthoquinone.**

Table S2. Free binding energy results (in kcal.mol^{-1}) from molecular docking calculations of compounds **5**–**7**, the two regioisomers of compound **8**, Paracetamol (PCM) and *N*-arachidonoylphenolamine (AM404) with proteins TRPV1 receptor (3J5P) and CB1 receptor (5U09).

**Compound**	**Isomer**	**ΔE_{Bind} (kcal.mol^{-1})**	
		**TRPV1 receptor**	**CB1 receptor**
		**3J5P**	**5U09**
**5**	–	−9.2	−9.0
**6**	–	−8.7	−9.0
**7**	–	−9.1	−8.9
	8-nitro (minor)	−9.3	−9.0
**8***	5-nitro (major)	−9.6	−9.1
**PCM**	–	−5.9	−6.1
**AM404**	–	−8.9	−9.1

Docking analyses involving TRPV1 and CB1 receptors are presented for exploratory purposes only. These results are considered highly speculative and do not constitute evidence of functional target engagement or mechanistic involvement. No experimental validation of these interactions is provided in the present study.

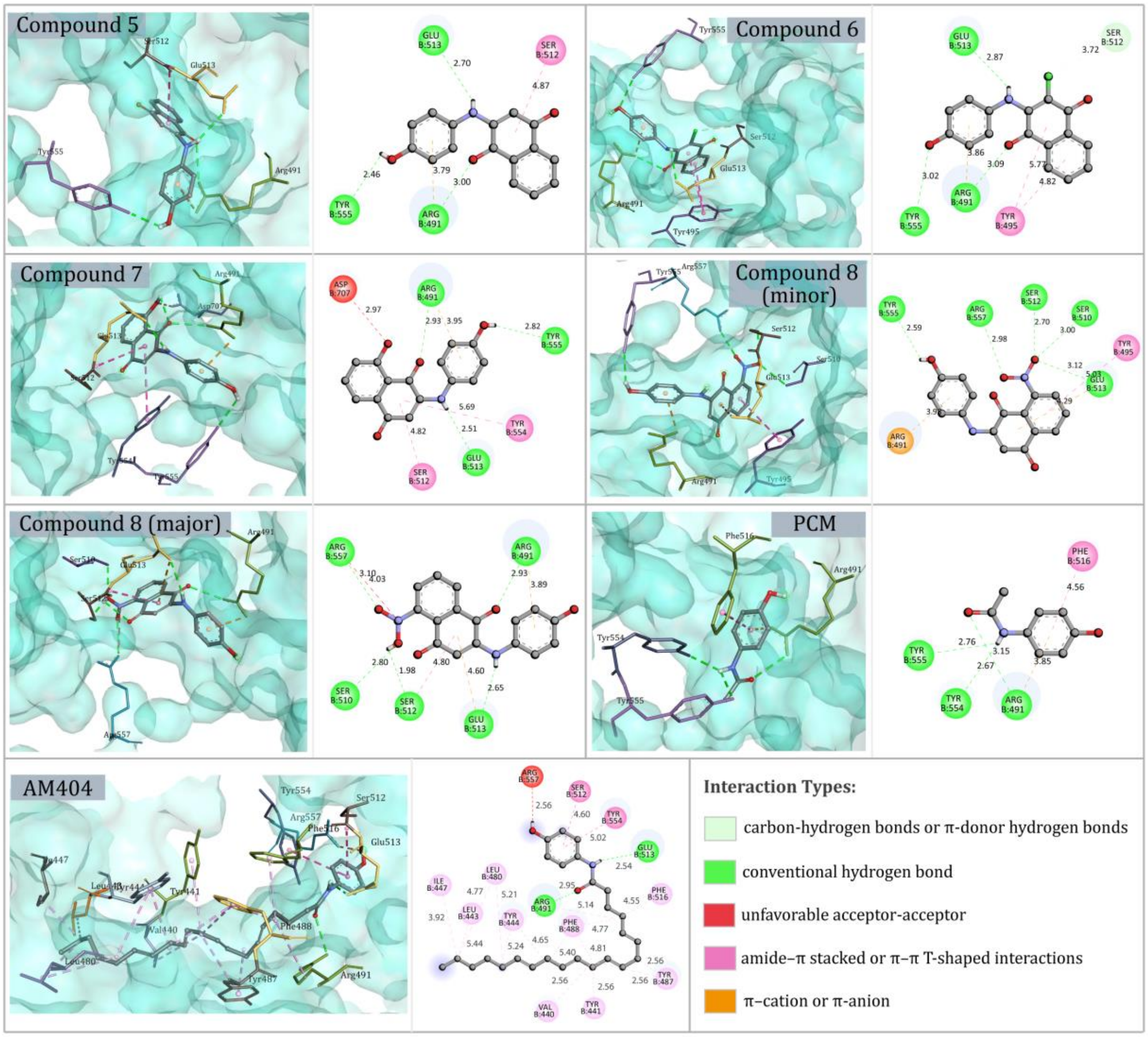

Figure S14. Representative docking poses and interaction profiles of compounds **5**–**7**, the two regioisomers of compound **8**, paracetamol (PCM), and *N*-arachidonoylphenolamine (AM404) within the TRPV1 receptor binding site.

The left panels show the overall receptor structure with the ligand-binding region highlighted, whereas the middle panels depict a magnified view of the binding pocket, illustrating the ligand orientation and key interacting residues. Hydrogen atoms are omitted in some cases for the sake of clarity.

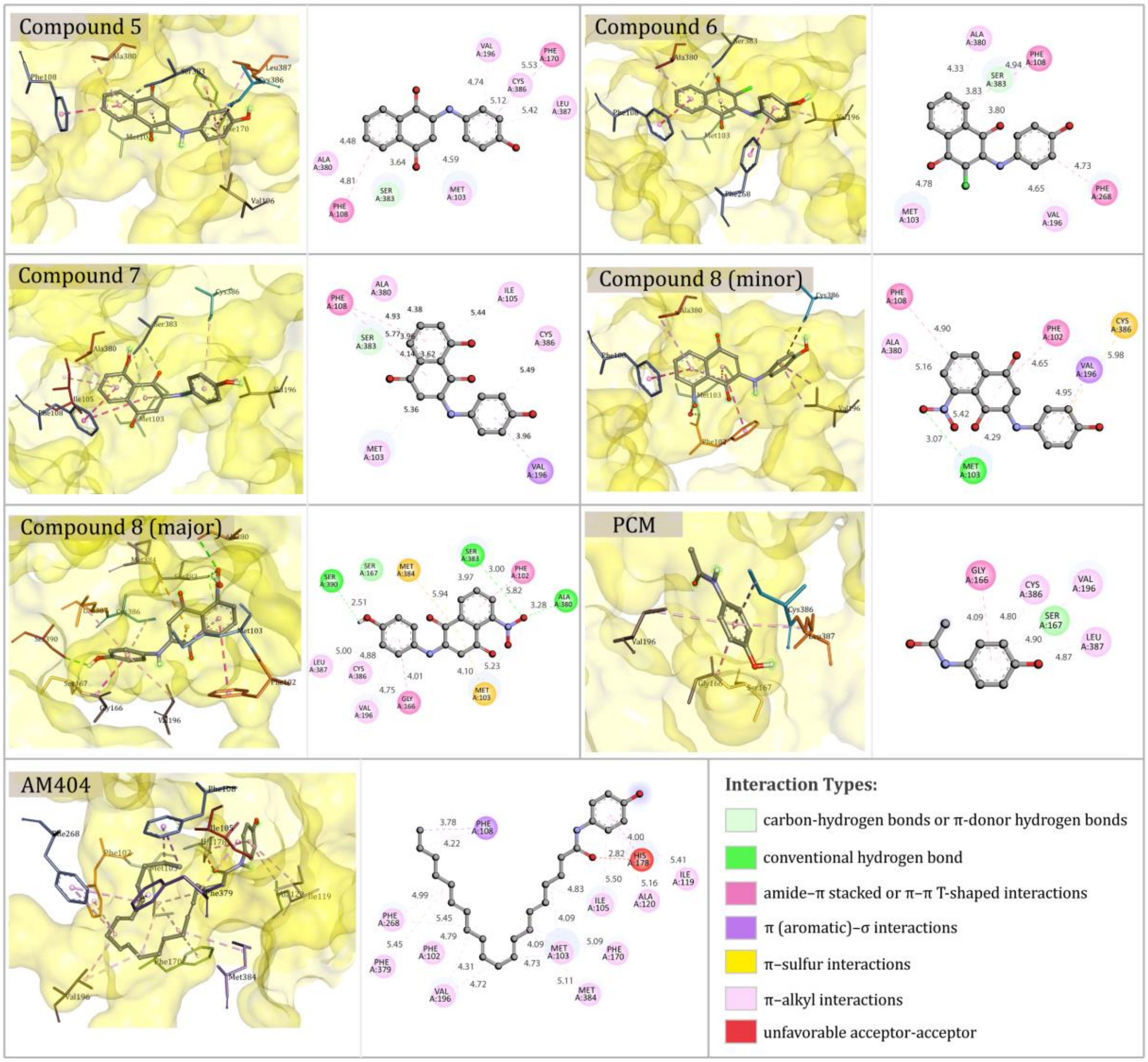

Figure S15. Representative docking poses and interaction profiles of compounds **5–7**, the two regioisomers of compound **8,** paracetamol (PCM), and *N*-arachidonoylphenolamine (AM404) within the CB1 receptor-binding site.

The left panels show the overall receptor structure with the ligand-binding region highlighted, whereas the middle panels depict a magnified view of the binding pocket, illustrating the ligand orientation and key interacting residues. The right panel presents two-dimensional interaction diagrams summarizing the ligand–residue contacts. Hydrogen atoms are omitted in some representations for the sake of clarity.

Cartesian Coordinates

APAP

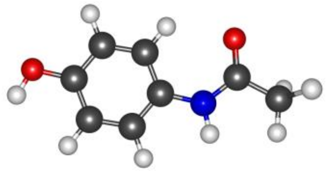

6	-3.969109000	0.484433000	-0.000049000
6	-2.616665000	-0.203809000	0.000070000
8	-2.525986000	-1.426575000	-0.000011000
7	-1.542565000	0.643021000	0.000028000
6	-0.161519000	0.335860000	0.000017000
6	0.739589000	1.408333000	0.000007000
6	2.112351000	1.190569000	-0.000001000
6	2.613632000	-0.113051000	-0.000008000
6	1.721411000	-1.185696000	-0.000002000
6	0.346724000	-0.970768000	0.000011000
8	3.952114000	-0.395994000	-0.000012000
1	-4.524927000	0.156547000	-0.881113000
1	-3.915208000	1.573439000	0.000072000
1	-4.525164000	0.156353000	0.880790000
1	-1.756234000	1.629324000	0.000031000
1	0.365663000	2.426864000	0.000010000
1	2.791693000	2.036547000	-0.000003000
1	2.114425000	-2.195675000	-0.000008000
1	-0.336474000	-1.805024000	0.000018000
1	4.456659000	0.425801000	-0.000086000

C5

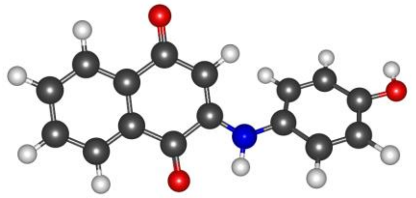

8	1.203149000	-2.441966000	0.200471000
6	1.389357000	-1.238565000	0.099024000
6	0.197902000	-0.308747000	0.008608000
7	-0.969216000	-0.988347000	0.071737000
6	-2.301417000	-0.522378000	0.049936000
6	-2.703278000	0.664476000	0.671388000
6	-4.038984000	1.054796000	0.646447000
6	-4.996190000	0.258339000	0.014435000
8	-6.318036000	0.587906000	-0.042095000
6	-4.602917000	-0.939993000	-0.589953000
6	-3.270018000	-1.321431000	-0.574451000
6	0.383473000	1.039083000	-0.145049000
6	1.692997000	1.644034000	-0.214315000
6	2.891887000	0.750945000	-0.086175000
6	4.172769000	1.298188000	-0.121307000
6	5.291349000	0.474645000	-0.012566000
6	5.137969000	-0.905761000	0.129530000
6	3.863810000	-1.462060000	0.163507000
6	2.738733000	-0.638448000	0.057665000
8	1.840630000	2.858160000	-0.377405000
1	-0.837404000	-1.995069000	0.077987000
1	-1.988205000	1.276532000	1.203165000
1	-4.335465000	1.977876000	1.133095000
1	-6.466769000	1.424492000	0.414453000
1	-5.349710000	-1.557838000	-1.073515000
1	-2.970951000	-2.246805000	-1.053784000
1	-0.450446000	1.719590000	-0.242723000
1	4.275126000	2.369870000	-0.235268000
1	6.284199000	0.908243000	-0.039331000
1	6.009468000	-1.543839000	0.213264000
1	3.724068000	-2.530161000	0.272385000

C6

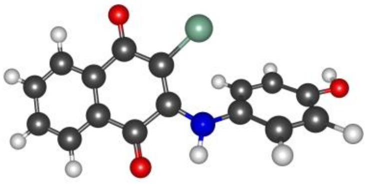

8	1.238797000	-2.603822000	-0.260663000
6	1.453725000	-1.405557000	-0.175844000
6	0.279030000	-0.447510000	-0.297913000
7	-0.887733000	-1.118104000	-0.411412000
6	-2.223503000	-0.722216000	-0.133276000
6	-2.543845000	0.074058000	0.968691000
6	-3.870565000	0.381217000	1.249542000
6	-4.894423000	-0.120754000	0.441663000
8	-6.212615000	0.141128000	0.669942000
6	-4.577714000	-0.934641000	-0.650438000
6	-3.251732000	-1.229292000	-0.934799000
6	0.525547000	0.902751000	-0.348154000
6	1.846223000	1.485943000	-0.126881000
6	2.985132000	0.540206000	0.101395000
6	4.259310000	1.053224000	0.336796000
6	5.338156000	0.190379000	0.519098000

C7

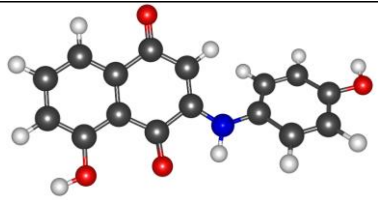

8	1.072964000	-2.248062000	0.119495000
6	1.235218000	-1.037915000	0.049589000
6	-0.005686000	-0.158176000	-0.012418000
7	-1.133526000	-0.899725000	0.045036000
6	-2.488669000	-0.504930000	0.039438000
6	-2.950525000	0.641543000	0.694208000
6	-4.304814000	0.962349000	0.681671000
6	-5.220910000	0.135238000	0.028705000
8	-6.558244000	0.398656000	-0.017934000
6	-4.767675000	-1.024536000	-0.607779000
6	-3.416448000	-1.336361000	-0.604289000
6	0.096933000	1.199158000	-0.141003000
6	1.368662000	1.867225000	-0.204556000
6	2.617792000	1.035116000	-0.094980000
6	3.840443000	1.689129000	-0.121065000
6	5.024205000	0.955556000	-0.030243000

6	5.152588000	-1.191759000	0.462162000	6	4.984621000	-0.423559000	0.081724000
6	3.884504000	-1.711890000	0.225782000	6	3.758166000	-1.104157000	0.105715000
6	2.799335000	-0.848932000	0.049471000	8	3.726982000	-2.449204000	0.212059000
8	2.041062000	2.696219000	-0.143730000	6	2.548280000	-0.374634000	0.020107000
1	-0.735121000	-2.117719000	-0.517564000	8	1.456023000	3.090912000	-0.346512000
1	-1.760375000	0.445448000	1.616831000	1	-0.943064000	-1.897700000	0.032094000
1	-4.110198000	1.001798000	2.106476000	1	-2.266488000	1.275456000	1.241121000
1	-6.303737000	0.701371000	1.449858000	1	-4.647871000	1.854766000	1.194163000
1	-5.377346000	-1.323368000	-1.269009000	1	-6.746755000	1.216987000	0.456641000
1	-3.006796000	-1.852442000	-1.787108000	1	-5.482578000	-1.667166000	-1.107036000
1	4.390521000	2.126926000	0.369442000	1	-3.070690000	-2.231720000	-1.108599000
1	6.325683000	0.596511000	0.703337000	1	-0.774735000	1.833102000	-0.221015000
1	5.993559000	-1.859752000	0.603273000	1	3.850272000	2.766543000	-0.213633000
1	3.718581000	-2.780616000	0.180441000	1	5.981746000	1.462542000	-0.048024000
17	-0.731130000	2.054647000	-0.754368000	1	5.903360000	-0.996389000	0.151415000
				1	4.632138000	-2.783050000	0.261810000
**C8-5NO2**				**C8-8NO2**			
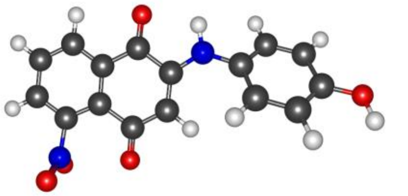				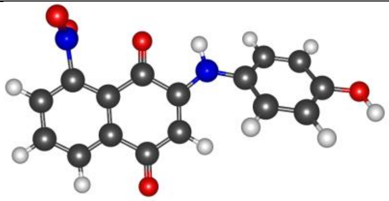			
8	0.142078000	2.930197000	-0.163006000	8	0.971842000	-1.878532000	-0.097400000
6	0.495053000	1.765469000	-0.088498000	6	0.964295000	-0.659931000	-0.052222000
6	-0.542801000	0.672013000	-0.009696000	6	-0.351530000	0.080881000	-0.050166000
7	-1.797495000	1.165924000	-0.053753000	7	-1.399604000	-0.769869000	-0.018045000
6	-3.041307000	0.496480000	-0.038271000	6	-2.786777000	-0.502000000	0.005621000
6	-3.256125000	-0.719706000	-0.694568000	6	-3.342814000	0.551436000	0.738216000
6	-4.512803000	-1.316368000	-0.674074000	6	-4.720429000	0.746644000	0.752824000
6	-5.576089000	-0.700442000	-0.009907000	6	-5.563031000	-0.116201000	0.048142000
8	-6.826956000	-1.237156000	0.046206000	8	-6.917960000	0.024803000	0.023714000
6	-5.371009000	0.527180000	0.628414000	6	-5.012569000	-1.184670000	-0.667179000
6	-4.115914000	1.115555000	0.615630000	6	-3.639089000	-1.371423000	-0.689353000
6	-0.154500000	-0.635348000	0.123481000	6	-0.377477000	1.448617000	-0.117525000
6	1.224554000	-1.041306000	0.181615000	6	0.820152000	2.245802000	-0.172310000
6	2.280463000	0.015550000	0.021258000	6	2.149080000	1.540973000	-0.104390000
6	3.640208000	-0.287538000	0.010321000	6	3.319720000	2.294773000	-0.131967000
6	4.624094000	0.690821000	-0.072960000	6	4.561340000	1.670284000	-0.077755000
6	4.247168000	2.027503000	-0.162791000	6	4.645045000	0.282333000	0.011932000
6	2.899587000	2.367853000	-0.166356000	6	3.473116000	-0.457012000	0.050109000
6	1.925460000	1.372500000	-0.073724000	6	2.209408000	0.139823000	-0.016032000
8	1.560492000	-2.212064000	0.369998000	8	0.790300000	3.473828000	-0.269185000
1	-1.825018000	2.180859000	-0.041857000	1	-1.124714000	-1.744003000	-0.094860000
1	-2.458780000	-1.194512000	-1.249455000	1	-2.713331000	1.206751000	1.324370000
1	-4.666798000	-2.258733000	-1.188762000	1	-5.139249000	1.566589000	1.326638000
1	-6.845066000	-2.076776000	-0.428386000	1	-7.177623000	0.788614000	0.552435000
1	-6.200699000	1.004064000	1.135648000	1	-5.671205000	-1.854888000	-1.205793000
1	-3.961371000	2.062407000	1.120526000	1	-3.217772000	-2.195537000	-1.253967000
1	-0.875795000	-1.432968000	0.229388000	1	-1.308233000	1.995998000	-0.159053000
1	5.669344000	0.408517000	-0.073125000	1	5.470098000	2.258411000	-0.103600000
1	5.006752000	2.795610000	-0.231262000	1	5.604564000	-0.216187000	0.061487000
1	2.585764000	3.400928000	-0.238376000	1	3.235973000	3.371218000	-0.201309000
7	4.128854000	-1.689235000	0.067698000	7	3.631855000	-1.925745000	0.195398000
8	4.174666000	-2.304710000	-0.983728000	8	3.480308000	-2.393787000	1.310668000
8	4.531711000	-2.092208000	1.146630000	8	3.966148000	-2.547517000	-0.799732000

Figure S16. The optimized structures of compounds **5–7**, the two regioisomers of compound **8**, and the reference drug paracetamol (APAP)

Table S3. **Dose-averaged raw pharmacological values used for normalization.**

Values correspond to the arithmetic mean of the two tested doses (12.5 and 25 mg/kg) for each compound.

Compound	Writhing reduction (%)	Edema AUC reduction (%)	Hot plate increase (%)	Temperature AUC reduction (%)
5	53.95	47.90	231.00	0.00
6	61.30	41.25	106.90	0.40
7	77.55	50.75	125.85	0.30
8	85.35	43.55	200.00	0.20

Table S4. Min–Max Normalized Integrated Pharmacological Parameters (0–1) of compounds **5**–**8**.

Compound	PERnorm	AInorm	CENnorm	APYnorm	Global Score
**5**	0.00	0.70	1.00	0.00	**0.43**
**6**	0.23	0.00	0.00	1.00	**0.31**
**7**	0.74	1.00	0.15	0.75	**0.66**
**8**	1.00	0.24	0.75	0.50	**0.62**

PER_{norm}= Normalized Peripheral Antinociceptive Efficacy; AI_{norm}= Normalized Anti-Inflammatory Activity; CEN_{norm}= Normalized Central Antinociceptive Activity; APY_{norm}= Normalized Antipyretic Activity. Integrated pharmacological parameters normalized to a 0–1 scale using min–max transformation across compounds. Endpoint values correspond to dose-averaged percentages for peripheral antinociception (writhing inhibition), anti-inflammatory activity (edema AUC reduction), central antinociception (hot plate AUC increase), and antipyretic activity (temperature AUC reduction). Global score represents the mean of the four normalized parameters.

Normalization was performed using min–max scaling:

$$X_{norm} = \frac{X_i - X_{min}}{X_{max} - X_{min}}$$
